# Enhanced hydrogen adsorption on boron nickel gold modified Si60 nanocluster via DFT and machine learning analysis

**DOI:** 10.1038/s41598-026-52240-0

**Published:** 2026-05-19

**Authors:** Onyinye J. Ikenyirimba, Gideon E. Mathias, Chukwuma C. Nwanazoba, Anthony C. Iloanya, Valentine Chikaodili Anadebe, Eno E. Ebenso

**Affiliations:** 1 Department of Chemistry and Biochemistry, University of Arizona, Tucson, AZ 85721 USA; 2 Department of Pure and Applied Chemistry, Faculty of Physical Sciences, University of Calabar, P.M.B 1115, Calabar, Nigeria; 3 Biomedical Computational Chemistry Research Group, Lagos, Nigeria; 4 Department of Chemical Engineering, Auburn University, Alabama, 36849 USA; 5 Department of Chemical Engineering, Faculty of Engineering, Nnamdi Azikiwe University, P.M.B 5025, Awka, Nigeria; 6 Department of Physics, Lehigh University, Pennsylvania, USA; 7 Centre for Materials Science, College of Science, Engineering and Technology, University of South Africa, Johannesburg, 1710 South Africa

**Keywords:** Hydrogen evolution reaction (HER), Silicon-based nanoclusters, Doping techniques, Hydrogen adsorption, Catalytic efficacy, Chemistry, Materials science, Nanoscience and technology

## Abstract

In this study, we investigated the catalytic potential of silicon-based nanoclusters, specifically Si_**60**_, for hydrogen evolution reactions (HER), focusing on enhancements via substitutional doping with nickel (Ni) and boron (B), where boron and nickel are incorporated into the Si₅₉ lattice, enabling electronic structure modulation. Using Density Functional Theory (DFT) with the B3LYP functional, we examined hydrogen adsorption behavior on boron- doped, Ni-doped, gold-encapsulated Si₅₉ nanoclusters, denoted as B_x_^dop^Ni^dop^Au^enc^Si₅₉ (x = 1, 2, 3). Our findings show that doping introduces minimal structural distortion while improving cluster stability and reactivity. Boron-doping notably reduces the energy gap, enhancing electron transfer and promoting hydrogen adsorption. Gibbs free energy analyses confirm somewhat favorable catalytic activity, with ΔG_H_ values ranging from − 0.837 to − 0.848 eV, highlighting the B_x_^dop^Ni^dop^Au^enc^Si₅₉ engineered doped nanoclusters as promising catalysts. Additionally, machine learning models, particularly ElasticNet Regression (R^2^ = 0.9942), accurately predict hydrogen adsorption energies across various surfaces, from pristine H₂@Si₆₀ to B₃- doped systems. This demonstrates the models’ capability to capture structure–property relationships, accelerating catalyst optimization. Computational screening of Si_**59**_-based nanostructures revealed that H₂@B_2_^dop^Ni^dop^Au^enc^Si₅₉ exhibits a ΔG_H_ value of − 0.836 eV, suggesting comparatively balanced hydrogen adsorption and improved HER catalytic potential among the evaluated systems. Overall, the results suggest that precise doping and surface engineering can significantly enhance the electronic, storage, and catalytic properties of silicon nanoclusters, offering valuable insights for the design of efficient HER catalysts in future sustainable energy technologies.

**Supplementary Information:**

The online version contains supplementary material available at 10.1038/s41598-026-52240-0.

## Introduction

The escalating global demand for energy, coupled with the urgent imperative to combat climate change, calls for a fundamental shift toward clean, sustainable energy systems. Our heavy reliance on fossil fuels has led to alarming environmental issues, including greenhouse gas emissions and air pollution, while also threatening long-term energy security^1^. This stark reality underscores an urgent need to develop and adopt innovative energy technologies that are environmentally sustainable and economically feasible, securing our future against the mounting ecological and geopolitical challenges. Hydrogen stands out among potential sustainable energy carriers due to its exceptional energy density per unit mass and its environmentally benign combustion, producing only water^2^. With a weight-specific energy density of approximately 33.3 kWh/kg, substantially higher than gasoline at 12.2 kWh/kg, hydrogen offers immense promise for diverse applications, from transportation to power generation^3^. However, harnessing hydrogen’s potential is severely constrained by formidable storage obstacles. Its small molecular size and low-density complicate containment and safety. Hitherto, compressed gas, liquefaction, and metal hydrides present significant limitations in storage methods. Storing hydrogen as a compressed gas at pressures up to 10,000 psi requires robust, heavy tanks, which reduce vehicle efficiency and increase costs^4^. The energetic cost of compressing hydrogen is also substantial. Liquefying hydrogen, while providing higher volumetric density, demands cryogenic temperatures (~ − 253 °C), requiring significant energy input and sophisticated insulation, as boil-off losses further diminish the overall storage efficiency^5^.

Tentatively, studies have shown that metal hydrides offer an alternative that is safer and more compact, but many suffer from low gravimetric capacity and slow absorption/desorption kinetics^6^. Additionally, some metal hydrides require high temperatures to release hydrogen, impacting their practicality. Developing advanced hydrogen storage and catalytic materials with higher energy densities, improved safety, and cost-effectiveness is crucial for enabling hydrogen to fulfill its role as a versatile, clean energy carrier. Success in this area could unlock its use across transportation, electricity generation, and industrial processes such as ammonia synthesis and steel manufacturing. Hitherto, in the quest for innovative hydrogen storage solutions and potential HER catalytic material, nanostructured materials, particularly silicon nanoclusters, have emerged as up-and-coming candidates. Silicon-based optimized nanoclusters represent a highly promising catalyst for the Hydrogen Evolution Reaction (HER) due to their exceptional combination of activity, stability, and sustainability^6^. Their unique nanostructure offers an extremely high surface area and the ability to finely tune the electronic structure and local coordination environment through substitutional doping and controlled structural modification of the silicon framework. This allows for enhanced hydrogen adsorption, faster reaction kinetics, and improved overall catalytic efficiency. Importantly, silicon is one of the most abundant and environmentally friendly elements on Earth, making these nanoclusters a cost-effective and scalable solution for hydrogen production. In stark contrast, traditional HER catalysts such as platinum, palladium, and other noble metals, while highly active, suffer from critical limitations^7^. They are prohibitively expensive, scarce, and face issues of deactivation over time due to poisoning and corrosion. These factors significantly hinder mass deployment and increase the overall cost of hydrogen generation. Noble metals also require complex synthesis processes, which add to their economic and environmental footprint.

Thus, silicon-based nanoclusters not only promise comparable or superior catalytic performance but also offer a sustainable, affordable, and scalable pathway toward clean hydrogen energy. Embracing these materials could revolutionize hydrogen production, making it more accessible and economically viable, and accelerating the transition to a sustainable energy future^7,8^. Their unique properties, derived from their nanoscale dimensions, include a high surface area-to-volume ratio and the ability to tailor their electronic properties. These features significantly influence their interactions with hydrogen molecules, potentially making them highly effective for storage applications. More so, the increased surface area of silicon nanoclusters provides numerous adsorption sites for hydrogen, potentially increasing storage capacity^9,10^. Additionally, the ability to modify their electronic structure offers opportunities to optimize the binding energy, enhancing both storage and release processes. Silicon, being the second most abundant element in the Earth’s crust, is readily available and cost-effective. Its abundance ensures a sustainable supply chain, while its relatively low cost compared to other advanced materials makes it an attractive option for large-scale energy storage solutions^11^. Research into silicon nanoclusters aims to leverage these advantages to develop practical, scalable hydrogen storage technologies. Despite the intrinsic advantages of silicon nanoclusters, their pristine forms encounter substantial barriers to efficient hydrogen adsorption. A key challenge lies in their low reactivity with gas molecules, predominantly due to surface passivation caused by silicon oxide layers. Silicon’s tendency to oxidize rapidly in ambient environments results in the formation of this oxide barrier, which obstructs direct interaction between hydrogen molecules and the silicon surface^11^. Consequently, the number of active adsorption sites diminishes, and the interaction strength weakens. Additionally, the presence of oxygen modifies the silicon’s electronic band structure, potentially creating energy levels unfavorable for hydrogen binding. Pristine silicon nanoclusters also suffer from stability issues under certain conditions, as ongoing oxidation can undermine their structural integrity and long-term viability for hydrogen storage and catalytic ability^9,12–14^. While silicon naturally shows an affinity for hydrogen, issues related to oxidation susceptibility and interaction stability hinder its practical application in hydrogen storage technologies. To overcome these limitations, researchers are exploring various modification strategies, including doping with transition metals and surface engineering^15,16^. These approaches aim to tailor silicon’s properties at the nanoscale to promote better hydrogen adsorption. Doping silicon nanoclusters with transition metals, such as titanium, introduces active sites capable of stronger interaction with hydrogen molecules. Transition metal atoms act as catalytic centers, facilitating hydrogen dissociation into atomic form and boosting binding energy via mechanisms like spillover effects. Nonetheless, optimizing these modifications requires addressing challenges such as dopant atom agglomeration, which can reduce efficiency, and ensuring that active sites remain accessible to hydrogen. Achieving a uniform distribution of dopants within the nanoclusters is crucial for maximizing storage performance and ensuring consistent catalytic activity across the material^17^. The automotive industry is shifting toward electric and hydrogen fuel cell vehicles (FCEV) to cut carbon emissions. While battery tech has advanced, challenges like limited range, long charging times, and heavy batteries hinder long-distance and large vehicles. FCEVs offer longer ranges and quick refueling but require lightweight, efficient hydrogen storage^18^. Engineered silicon nanoclusters, with surface modifications, could provide high-capacity, stable storage solutions, enabling long-haul trucks and family SUVs to travel farther with minimal downtime^19^. Overcoming storage limitations is key to enabling sustainable, efficient transportation at scale.

Existing hydrogen storage methods and catalytic materials for hydrogen production often rely on bulky, heavy, or energy-intensive systems, which pose significant obstacles for practical vehicle applications. This study investigates engineered silicon nanoclusters modified through substitutional doping with transition metals such as nickel and gold, enabling precise modulation of their electronic structure to achieve enhanced stability, hydrogen storage capacity, and catalytic performance. Using density functional theory simulations, we aim to understand their interaction with hydrogen, providing insights to develop safe, scalable storage and catalytic solutions. Herein, we demonstrate that a stepwise strategy integrating encapsulation and substitutional doping significantly enhances the catalytic performance of silicon-based materials for hydrogen production, advancing sustainable energy technologies. Our findings underscore the effectiveness of these strategies in boosting the catalytic performance of these synergistically optimized nanostructure materials. To the best of our knowledge, systematic studies exploring the HER activity and hydrogen storage capabilities of these engineered nanostructured materials remain scarce. Herein, we employ targeted density functional theory (DFT) calculations integrated with regression analysis to elucidate their catalytic behavior. Recent advances in machine learning (ML) have significantly transformed catalyst discovery by enabling data-driven identification of structure–property relationships and rapid screening of candidate materials. In catalysis, descriptor-based approaches, particularly those centered on the Gibbs free energy of hydrogen adsorption (ΔG_H_), have been widely established as effective predictors of hydrogen evolution reaction (HER) activity, Jens K. Nørskov et al.^20^. The integration of ML with density functional theory (DFT) has further accelerated this process by allowing efficient exploration of large compositional and configurational spaces, reducing the reliance on computationally expensive first-principles calculations, Zachary W. Ulissi et al.^21^; Keith T. Butler et al.^22^. These approaches have been successfully applied in the discovery and optimization of catalytic materials, where ML models are used not only to predict adsorption energies but also to identify key descriptors and guide rational catalyst design^21^. Despite these advances, the combined application of ML and DFT in engineered silicon nanoclusters remains underexplored, motivating the present study.

This study aims to deliver theoretical insights and a valuable guide that informs the rational design and synthesis of optimized silicon-based nanocluster materials for efficient hydrogen production.

## Computational framework

In this study, the hydrogen evolution reaction (HER) mechanism was explicitly considered by modeling both the Volmer–Heyrovsky and Volmer–Tafel pathways, which are well-established routes for HER in acidic media. These mechanisms involve key steps: the initial proton-electron transfer forming adsorbed hydrogen (H*) on the catalyst surface (Volmer step), followed either by electrochemical desorption forming H₂ (Heyrovsky step) or recombination of two adsorbed H* atoms (Tafel step). Furthermore, the inclusion of the H₂@catalyst structure was not intended to suggest a distinct HER intermediate but rather to capture the post-reaction state where molecular hydrogen is stabilized on the surface before desorption. This approach enables a detailed assessment of hydrogen binding strength and the desorption energy barrier, both of which are essential for evaluating HER catalytic performance. Thus, the mechanistic framework and associated intermediates presented are consistent with standard computational protocols in HER studies, as presented in the subsequent section of this work. The hydrogen adsorption behavior on various configurations of the Si₅₉ nanocluster was investigated, specifically focusing on three distinct structures: B-doped (B_**2**_^**dop**^Ni^**dop**^Au^**enc**^Si₅₉), Ni-doped (Ni^**dop**^Au^**enc**^Si₅₉), and Au-encapsulated (Au^**enc**^Si_60_) nanoclusters. Our analysis was conducted using Density Functional Theory (DFT) via the Gaussian 16 software package^23^, a well-established quantum–mechanical approach that enables accurate calculations of the electronic structure of molecules and materials, making it particularly suitable for examining hydrogen adsorption phenomena.

For our calculations, we employed the B3LYP hybrid functional as the exchange–correlation functional^24,25^. B3LYP combines Becke’s three-parameter exchange functional with the Lee–Yang–Parr correlation functional, and it is widely recognized for its accuracy in modeling both structural and electronic properties of nanomaterials. To effectively describe the electronic states of the heavier elements in our system, namely nickel and gold, we utilized the Stuttgart/Dresden effective core potential (SDD) basis set^26^. This basis set simplifies the computational process by substituting the core electrons of these heavy atoms with an effective core potential (ECP), allowing us to focus computational resources on the valence electrons while maintaining high accuracy. More so, the B3LYP hybrid functional was employed to describe exchange–correlation effects, owing to its demonstrated accuracy in modeling nanomaterials and transition-metal systems. To effectively describe the heavier atoms involved, namely gold (Au), nickel (Ni), and boron (B), the Stuttgart/Dresden (SDD) effective core potential (ECP) basis set was used^26^, which simplifies the treatment of core electrons while maintaining high accuracy for valence electronic states. Vibrational frequency analyses confirmed the nature of the optimized structures, ensuring they correspond to true minima on the potential energy surface. The choice of this computational framework balances computational efficiency with the accuracy required for insightful mechanistic and electronic-structure interpretations in our study of HER catalysis. Vibrational frequency analyses were performed at the same theoretical level to confirm that all optimized structures correspond to true minima (no imaginary frequencies) and that all transition states exhibit exactly one imaginary frequency, verifying them as first-order saddle points.

Incorporating empirical dispersion corrections is crucial for accurately capturing non-covalent interactions, such as van der Waals forces, which significantly influence adsorption processes. To address this, we applied Grimme’s D3 correction with Becke-Johnson damping, ensuring a more realistic treatment of dispersion forces within the DFT framework^27^. All calculations were performed in the ground state, assuming the system’s most stable electronic configuration. This methodological choice enables a comprehensive evaluation of the interactions between hydrogen and the B_**x**_^**dop**^Ni^**dop**^Au^**enc**^Si₅₉ nanoclusters, where x = 1, 2, or 3 atomic boron, providing insights into binding energies, geometrical configurations, and electronic structure changes upon hydrogen adsorption.

We calculated the density of states (DOS), partial densities of states (PDOS), and total densities of states (TDOS) for the complexes using the Multiwfn software^28^, whereas the various adsorption sites were deciphered utilizing the molclus software^29^. Transition state (TS) calculations were conducted using the complete Linear Synchronous Transit/Quadratic Synchronous Transit (LST/QST) method, maintaining the same theoretical level as the ground state geometry optimization. Graphical representations of our findings were generated using Python with the Matplotlib library^30^. More so, charge transfer calculations and stabilization analyses were performed using the Natural Bond Orbital (NBO) and Mulliken methods. Additionally, we calculated the Gibbs free energy changes associated with the hydrogen evolution reaction (HER) activity, including the intermediate adsorption state (2H^*^) and the ground state H adsorption. The Gibbs free energy for the proton-electron combination G(H^**+**^ + e^**−**^) was derived as 1/2 G(H_**2**_) under standard conditions, consistent with the equilibrium potential. These calculations were performed at zero internal energy (U) = 0 V, hydrogen partial pressure (*P*_*H2*_) = 1 bar, pH = 0, and temperature = 298 K.

Furthermore, machine learning analyses were performed using Jupyter Notebook^30^, which facilitated the development and validation of predictive models for adsorption energies across various functionalized surfaces. This integration of computational and machine learning methodologies enhances our understanding of the catalytic properties of the engineered nanoclusters.

## Results and discussion

### Structural optimization

The geometry study on the various proposed-modified nanocluster surfaces reveals significant variations in bond lengths, which are crucial for understanding their catalytic properties. In the pristine Hydrogen-adsorbed surface examined, H₂@Si₆₀, the bond lengths between Si atoms (Si₂₃-Si₂₂) and between Si₂₂ and hydrogen (H₆₁) were measured at 2.269 Å and 2.394 Å, respectively, before any modifications such as encapsulation and doping. When gold (Au) was introduced to encapsulate the system, the bond length between Si₂₃ and Si₂₂ decreased slightly to 2.218 Å, while the bond length between Si₂₂ and H₆₁ remained unchanged at 2.394 Å in the H₂@Au^**enc**^Si₆₀ system. This reduction in bond length indicates a structural rearrangement, suggesting that the adsorption of hydrogen significantly influences the geometry of the system. Therefore, it is essential to enhance the strength of hydrogen adsorption from physisorption (weak, physical interactions) to chemisorption (strong, chemical bonding).

To achieve this enhancement, we introduced nickel (Ni) into the system, resulting in the H₂@NiᵈᵒᵖAuᵉⁿᶜSi₅₉ configuration. In this modified system, the bond length between Ni and hydrogen (Ni–H) decreased to 2.270 Å, while the bond length between Si and Ni (Si–Ni) measured approximately 2.218 Å. These measurements indicate stable coordination, which is vital for effective hydrogen adsorption. More so, to comprehensively assess the structural stability of the engineered silicon nanoclusters, additional stability indicators were also evaluated beyond bond length analysis. Specifically, the total optimized structural bond lengths were also calculated, offering insights into their overall energetic robustness. Vibrational frequency analyses were also performed to confirm the dynamic stability of these structures; the absence of imaginary frequencies indicates that the structures represent true minima on the potential energy surface. Moreover, structural integrity was supported by the preservation of symmetry and the stability of the centroid position, as detailed in Fig. 2. The combined evidence from energetic evaluations, vibrational analyses, and geometric considerations collectively confirms the structural stability of the modified nanoclusters, underpinning their potential effectiveness as catalysts in hydrogen evolution reactions.

Tentatively, in the second geometry with boron doping ;(H₂@B₁ᵈ^op^NiᵈᵒᵖAuᵉⁿᶜSi₅₉), a slight shift in bond lengths, with Ni–H measuring 2.414 Å and Ni-Si around 2.269 Å was observed. This suggests that the presence of boron doping induces electronic redistribution within the Si₅₉ framework, which modulates the Ni–H interaction strength and alters hydrogen adsorption energetics. The changes in bond lengths highlight how boron doping introduces localized electronic perturbations and modifies charge distribution around the Ni active site, which in turn affects hydrogen adsorption capabilities.

**Figure 1:**
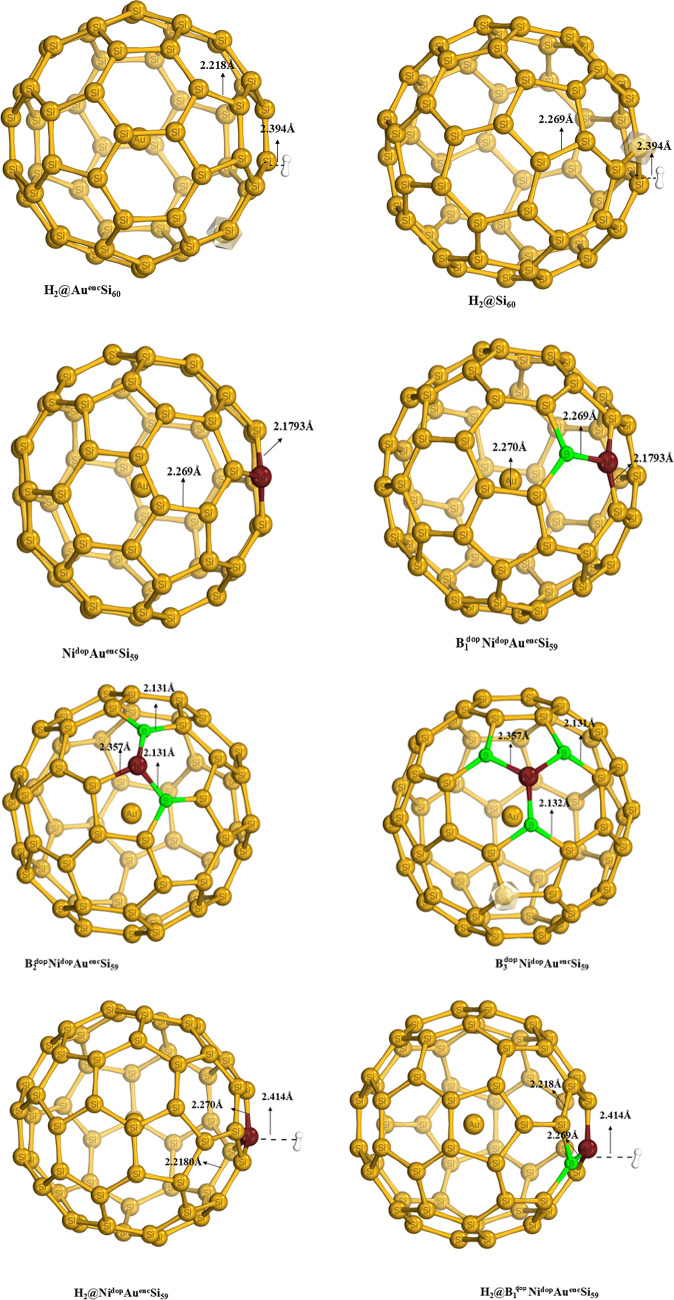

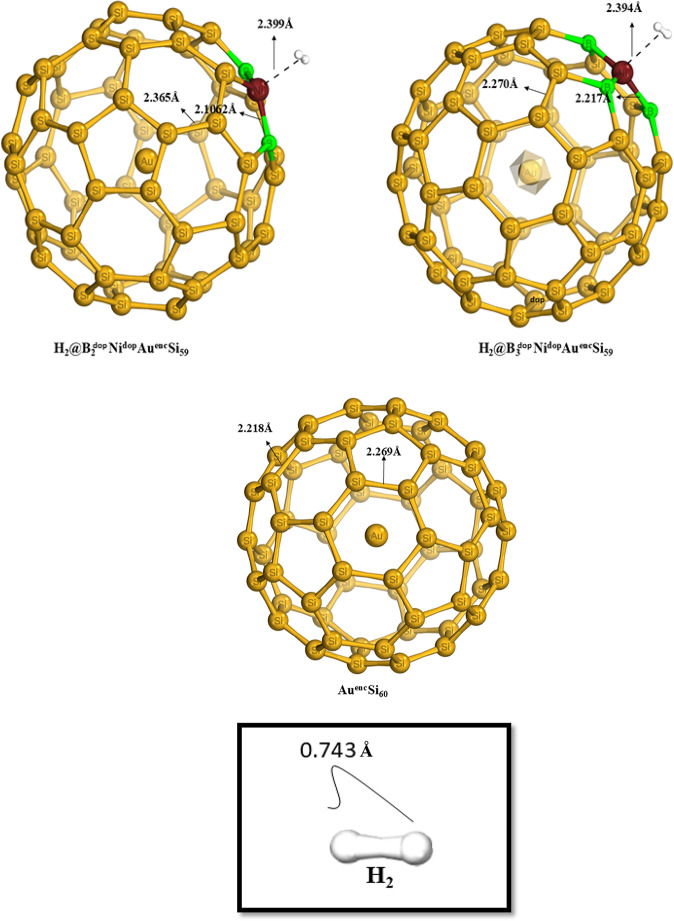
Graphical Depiction of Geometric Analyses for All Studied Engineered Silicon-Based Systems, Featuring Hydrogen-Adsorbed Surfaces and Molecular Hydrogen.

In furtherance, the analysis of bond lengths reveals subtle yet significant distortions upon hydrogen adsorption in both the Au-encapsulated Si₅₉ and the undoped Si₅₉ systems. Before hydrogen adsorption, the bond length between Si₂₂ and H₆₁ was 2.394 Å, with a slight difference of 2.2179 Å and 2.269 Å noted between Si₂₂ and Si₂₃ for both complexes. After hydrogen adsorption, the bond length between Si₂₂ and Si₂₃ showed minimal distortion, settling at 2.2180 Å for H₂@Ni^**dop**^Au^**enc**^Si₅₉ and 2.269 Å for H₂@B₁^**dop**^Ni^**dop**^Au^**enc**^Si₅₉. This indicates that hydrogen adsorption induces mild structural adjustments without significantly altering the local bonding configuration. Moreso, in the nickel-doped systems, both Ni^**dop**^Au^**enc**^Si₅₉ and B₁^**dop**^Ni^**dop**^Au^**enc**^Si₅₉ initially exhibited a consistent Ni-Si bond length of 2.21793 Å. However, following hydrogen adsorption, the bond length between Si₂₂ and Si₂₃ slightly increased, suggesting that the interaction with hydrogen leads to a mild expansion of the Si–Si framework. Additionally, upon boron doping, new Ni–B bonds were formed, measuring 2.131 Å between Ni₅₉ and B₆₀, and between Ni₅₉ and B₆₁ for the B_**2**_^**dop**^Ni^**dop**^Au^**enc**^Si₅₉ and B_**3**_^**dop**^Ni^**dop**^Au^**enc**^Si₅₉ surfaces, respectively, as shown in Fig. 1, respectively. This alteration induced by boron indicates that boron atoms help stabilize the structural framework while also modifying the local electronic environment, which further facilitates hydrogen adsorption.

These findings underscore the intricate relationship between hydrogen adsorption, boron- doped, and the resulting changes in local geometry. The adjustments in bond lengths upon modification, encapsulation, and doping highlight the potential of these modified systems as effective catalytic surfaces for hydrogen adsorption^31^. By enhancing the interaction strength and stabilizing the structural framework, these systems demonstrate improved catalytic properties, making them promising candidates for applications in hydrogen storage and conversion technologies.

#### Effects of encapsulation and co-doping on the stability of engineered Si₅₉ surfaces

In this work, boron incorporation occurs through substitutional integration into the Si₅₉ nanocluster framework. This modification is therefore more accurately described as doping rather than surface decoration^26^. Unlike decoration, which typically involves adsorption of species onto the surface, doping alters the lattice structure and electronic properties of the host material. The incorporated boron atoms modify the local electronic environment, thereby influencing charge distribution and catalytic activity. Herein, we investigate three primary forms of doping: endohedral (encapsulation), exohedral (intercalation), and co-doping^29^.

The catalytic activity of silicon-based structures, particularly Si_60_, can be significantly enhanced by the introduction of metal dopants such as nickel (Ni) and boron (B), as evidenced by previous research^31^. For example, studies have demonstrated that doping with Ni and B in silicon nanoclusters (Si₆₀) markedly improve their hydrogen evolution reaction (HER) activity; as it has been established that doping and co-doping Si nanoclusters with transition metals, example nickel (Ni) herein and heteroatoms, here, boron (B) enhances its catalytic activity for the hydrogen evolution reaction (HER) by altering its electronic structure^31,32^, with Ni introducing new electronic states that improve charge transfer and electron availability. The dopants create new active sites on the surface, facilitating reactant adsorption and product desorption, which increases the number of active sites for the HER. Co-doping leads to synergistic effects, optimizing reaction conditions as Ni boosts electronic conductivity and catalytic activity, while B enhances structural stability and modifies surface properties. Additionally, doping improves charge carrier mobility, leading to faster reaction kinetics by reducing recombination losses. To thoroughly evaluate and optimize the HER catalytic performance of Si₅₉ nanostructures, we analyze the specific impacts of doping, encapsulation, and doping on HER activity from a computational standpoint. In our computational approach, we employed the Hubbard correction to accurately model the metal doping process. Importantly, conformational geometry optimizations of the engineered Si_60_ nanostructures, incorporating encapsulation and doping with TM atoms, revealed no significant structural distortions. Specifically, the encapsulation of the gold (Au) atom induced only minimal microstrain, resulting in a slight positional shift of silicon atoms from their ideal configuration, with the Au atom substituting a silicon atom at an ultimate tensile strength of 0.4% compared to the bare Si₅₉ surface.

Furthermore, the doping of Si_60_ surfaces with Ni and B atoms did not lead to any adverse structural distortions, even in the presence of hydrogen adsorption. Instead, these modifications resulted in increased bond distances. The bare Si₅₉ surface is characterized by two hexagonal [r₆₆] facets and an outer pentagonal ring [r₆₅], with a computed Si–Si bond distance of 2.218 Å. We first explored molecular hydrogen adsorption at various predicted positions, utilizing the Molclus program^29^, including the centers of the pentagonal and hexagonal facets surrounding the doped atoms. After optimization, the most stable configuration for atomic hydrogen adsorption was identified. Hence, this configuration exhibited the lowest negative adsorption and dissociation energies, indicating that the engineered systems possess sufficient stability for catalytic activity testing.

**Figure 2:**
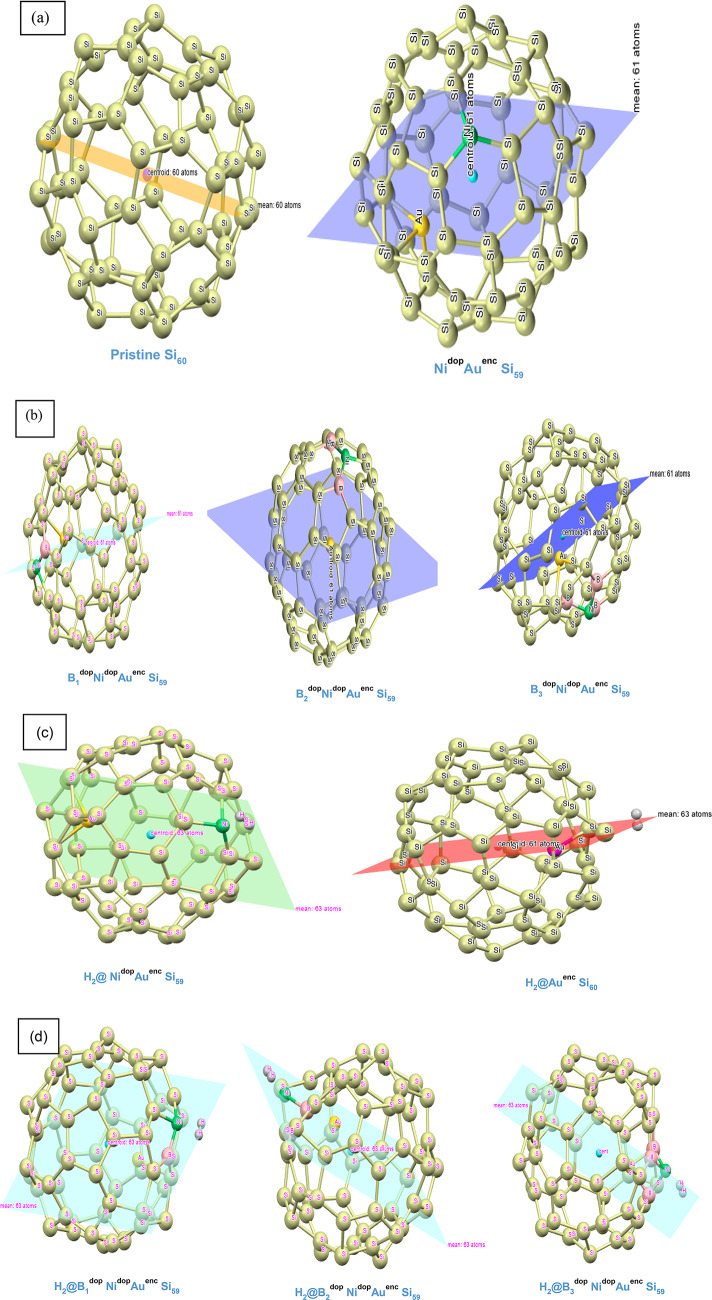
Centroid and Planes of the Studied Systems, illustrating the centroid and planes of the engineered Si₅₉ surfaces, highlighting the effects of boron-doped, gold encapsulation, and nickel doping on the structural characteristics of the systems.

More so, the calculation of the centroid of the Si_60_ nanostructure, as depicted in Fig. 2 below, is crucial as it provides a reference point for understanding the spatial distribution of atoms within the structure. In this study, the centroid was calculated based on the positions of 60 atoms, yielding a mean value of 60 for the pristine Si_60_ studied system. This calculation is significant because it allows us to assess the overall symmetry and balance of the nanostructure. The centroid serves as a geometric center, which is essential for evaluating how the introduction of dopants affects the overall geometry and electronic properties of the system.

By determining the centroid, we further analyzed how the distribution of atoms around this central point influences the stability and reactivity of the nanostructure. A well-defined centroid indicates a more stable configuration, which is critical for catalytic performance. The mean value of 60 suggests that the distribution of atoms is relatively uniform, which can enhance the interaction with adsorbates and improve catalytic activity.

Additionally, the calculated Gibbs free energy of adsorption (∆G_**ads**_) for the encapsulated, doped Si-based nanocluster systems is summarized in the results section. Notably, the encapsulation, and doping of the studied material with Ni and B resulted in more negative ∆G_**ads**_ values that approach zero, thereby supporting the HER catalytic activity of the engineered silicon-based nanocluster (Si_60_) system.

Inclusively, our findings highlight the significant role of encapsulation, doping, and co-doping in enhancing the stability and catalytic performance of Si_60_ surfaces. The enhanced catalytic performance observed in the boron and nickel co-doped systems can be attributed to the synergistic modification of the electronic structure and local coordination environment within the Si₅₉ framework. In particular, substitutional boron-doping introduces localized electronic perturbations, while nickel provides active sites that facilitate hydrogen adsorption and activation. Gold incorporation further contributes to structural stabilization and electronic modulation, collectively leading to improved hydrogen adsorption energetics and catalytic behavior.

Although this study is computational, the modeled Si₅₉-based nanoclusters, featuring substitutional boron and nickel doping along with gold incorporation, can be reasonably approximated using established experimental approaches. Silicon nanoclusters are commonly synthesized through bottom-up methods such as chemical vapor deposition (CVD), laser ablation, and magnetron sputtering, which allow for precise control over size and morphology at the nanoscale^33,34^. Vacancy-containing structures, such as Si₅₉, may arise during cluster growth or be introduced through post-synthetic treatments like ion irradiation or thermal processing^35^. The incorporation of boron and nickel into the silicon framework can be achieved either during synthesis (in situ doping) or via post-synthetic techniques such as ion implantation and thermal diffusion, both of which are widely used for introducing heteroatoms into silicon materials^35,36^. Similarly, gold incorporation can be realized through vapor-phase deposition, co-sputtering, or solution-based methods that promote strong metal–silicon interactions and structural stabilization^37,38^.

Taken together, these experimentally grounded strategies provide realistic pathways for approximating the systems modeled in this work and highlight the potential for translating the present computational insights into experimentally accessible catalytic materials for hydrogen evolution reactions^39^

### Descriptors for computational catalytic metrics

In this study, we analyzed the frontier molecular orbitals (FMO) and quantum descriptors, as detailed in Table 1, to provide a comprehensive understanding of the electronic and catalytic properties of the systems under investigation, particularly regarding hydrogen adsorption and the catalytic hydrogen evolution reaction (HER) potential. Table 1 provides a comprehensive overview of the electronic and quantum chemical properties of the studied silicon-based nanostructures, which are crucial for understanding their catalytic behavior in hydrogen evolution reactions (HER). The table includes various descriptors such as ionization potential (IP), electronegativity (χ), HOMO and LUMO energies, the energy gap (E_**g**_), and related parameters like chemical hardness (η), electrophilicity index (S), and the average chemical potential (μ). The ionization potential reflects the ease with which a system can donate electrons, indicating its potential reactivity, while the HOMO and LUMO energies give insights into the system’s ability to donate and accept electrons, respectively. A smaller energy gap (E_**g**_) often correlates with higher reactivity and better electrical conductivity, both desirable traits for catalysts involved in HER. Modifications such as gold encapsulation, boron and nickel doping, influence these descriptors significantly; for example, nanostructures with reduced HOMO–LUMO gaps and increased electrophilicity are more reactive and better suited for facilitating electron transfer during hydrogen adsorption and evolution. The percentage change in the energy gap (%ΔE_**g**_) highlights how structural alterations impact these electronic properties relative to the baseline Si₆₀ system. Collectively, these descriptors elucidate how modifications enhance or diminish the systems’ catalytic potential by affecting charge transfer capabilities, stability, and reactivity, thereby guiding the design of more efficient silicon-based catalysts for hydrogen production.

**Table 1:** Overview of frontier molecular orbital distribution, HOMO–LUMO energy gap, and quantum descriptors for analyzed systems.

Modeled Systems	IP/eV	E_a_(eV)	χ(eV)	HOMO/eV	LUMO/eV	E_g_(eV)	%ΔE_g_	ꞷ(eV)	η(eV)	μ (eV)	S(eV)
Si_60_	5.652	4.263	− 0.695	− 5.652	− 4.263	1.389	–	17.692	0.695	4.957	1.439
Au^enc^Si_60_	5.634	4.219	− 0.707	− 5.634	− 4.219	1.414	–	17.166	0.707	4.927	1.414
Ni^dop^Au^enc^Si_59_	5.616	4.367	− 0.625	− 5.616	− 4.367	1.249	–	38.349	0.624	4.992	1.600
B_1_^dop^Ni^dop^Au^enc^Si_59_	5.655	4.287	− 0.684	− 5.655	− 4.287	1.369	–	18.053	0.684	4.971	1.461
B_2_^dop^Ni^dop^Au^enc^Si_59_	5.303	4.549	− 0.377	− 5.303	− 4.549	0.754	–	32.170	0.377	4.926	2.651
B_3_^dop^Ni^dop^Au^enc^Si_59_	5.549	4.416	− 0.566	− 5.549	− 4.416	1.132	–	21.935	0.566	4.983	1.767
H-Interacted Systems											
H_2_@Si_60_	5.651	4.262	− 0.694	− 5.651	− 4.262	1.389	–	17.679	0.695	4.956	1.439
H_2_@Au^enc^Si_60_	5.667	4.238	− 0.714	− 5.667	− 4.238	1.429	1.061	17.170	0.714	4.953	1.399
H_2_@Ni^dop^Au^enc^Si_59_	5.605	4.346	− 0.629	− 5.605	− 4.346	1.259	0.800	19.670	0.629	4.976	1.588
H_2_@B_1_^dop^Ni^dop^Au^enc^Si_59_	5.653	4.249	− 0.702	− 5.653	− 4.249	1.404	2.556	17.455	0.702	4.950	1.424
H_2_@B_2_^dop^Ni^dop^Au^enc^Si_59_	5.647	4.264	− 0.691	− 5.031	− 4.264	0.767	1.724	17.762	0.691	4.955	1.446
H_2_@B_3_^dop^Ni^dop^Au^enc^Si_59_	5.507	4.357	− 0.575	− 5.507	− 4.357	1.150	1.678	21.143	0.575	4.932	1.738

Herein, the HOMO–LUMO energy gap serves as a critical indicator of reactivity and stability. The pristine Si₆₀ system exhibits a moderate energy gap of 1.389 eV, suggesting a balanced reactivity and structural stability. However, when the Si₆₀ surface is encapsulated with gold (Au), this gap slightly increases to 1.414 eV. This minor increase indicates a slight stabilization of the system due to the presence of the gold atom, which may reduce its catalytic potential while enhancing the system’s structural integrity.

In contrast, the introduction of nickel doping (Ni^dop^Au^enc^Si₅₉), as illustrated in rows 3 through 8 of Table 1, significantly reduces the HOMO–LUMO gap to 1.249 eV, indicating a marked increase in reactivity. This reduction implies that the system becomes more susceptible to electron transitions, which is advantageous for the HER, as it facilitates the adsorption of molecular hydrogen and promotes efficient electron transfer during the catalytic process. The addition of boron further amplifies this effect, with the B₂-doped system exhibiting the smallest gap of 0.754 eV, signifying a high level of reactivity and readiness for catalytic processes. This trend of decreasing energy gaps suggests that boron- doped surfaces are particularly promising for enhancing the HER activity of silicon-based systems.

The chemical hardness and softness values further elucidate this shift in reactivity. The base Si₆₀ system has a moderate hardness of 0.695 eV, indicating a relatively stable surface. However, upon nickel doping, the hardness decreases to 0.624 eV, reflecting an increased tendency for electron exchange. This enhanced softness (1.600 eV) improves the surface’s ability to interact with hydrogen, thereby boosting its catalytic efficiency. The further softening observed in boron- doped systems, particularly in B₂^dop^Ni^dop^Au^enc^Si₅₉ with a softness value of 2.651 eV, highlights the ease with which these surfaces can deform their electron clouds, facilitate hydrogen adsorption and improve catalytic performance. This trend toward increased softness and reactivity is crucial for the HER, where efficient adsorption and desorption of hydrogen are essential for maintaining high catalytic turnover.

Regarding electronegativity, which measures a system’s ability to attract electrons, nickel doping results in a slight decrease in this value, indicating a greater propensity for electron donation. These characteristics benefit the HER, as the surface must donate electrons to the adsorbed hydrogen for effective proton reduction. Boron doping does not significantly alter the electronegativity. Still, it maintains an optimal balance, ensuring that these surfaces can effectively engage in both electron donation and acceptance, an essential aspect of HER catalysis. Crucially, the electrophilicity index, which quantifies a system’s ability to accept electrons, further underscores the catalytic potential of these materials. The base Si₆₀ system exhibits a moderate electrophilicity of 17.692 eV, suggesting a moderate capacity to accept electrons. However, upon nickel doping, this value increased dramatically to 38.349 eV, indicating a substantial enhancement in the system’s ability to accept electrons during the HER process. This increased electrophilicity is vital for promoting the adsorption and activation of molecular hydrogen, as electron-rich surfaces facilitate the dissociation of hydrogen molecules and improve catalytic efficiency. The boron-doped systems maintain high electrophilicity, with values around 32.170 eV for B₂^dop^Ni^dop^Au^enc^Si₅₉, ensuring that these surfaces remain highly reactive and capable of engaging in the necessary electron transfer processes for HER.

The observed changes in electronic structure upon hydrogen adsorption further support the catalytic potential of these systems. For instance, the HOMO–LUMO gap of H₂@Ni^dop^Au^enc^Si₅₉ decreases to 0.629 eV upon hydrogen adsorption, indicating that the presence of hydrogen enhances the system’s reactivity and lowers the energy barrier for electron transfer. This observation suggests that hydrogen adsorption modifies the electronic properties of the surface, making it more conducive to catalytic activity. Similarly, the electrophilicity index of H₂@Ni^dop^Au^enc^Si₅₉ remains high after hydrogen adsorption, confirming that the system retains its ability to accept electrons, which is crucial for driving the hydrogen evolution process.

Thus, the combined analysis of the frontier molecular orbitals and quantum descriptors demonstrates that doping with boron and nickel significantly enhances the catalytic properties of Si₅₉-based systems. This is so because the doping of Si_60_-based systems with nickel results in a reduced HOMO–LUMO gap, indicating increased reactivity and facilitating easier electron transfer, which enhances catalytic efficiency for processes like the hydrogen evolution reaction (HER). Additionally, the analysis reveals a decrease in chemical hardness and an increase in softness, suggesting a greater propensity for electron exchange and improved interaction with reactants, such as molecular hydrogen. The electrophilicity index also rises significantly in nickel-doped systems, indicating enhanced electron-accepting ability, which is crucial for effective hydrogen adsorption. Changes in electronic structure upon hydrogen adsorption further confirm the catalytic potential of these modified systems. Overall, the analysis demonstrates that boron and nickel doping significantly improve the catalytic potential of Si₅₉-based systems, leading to better performance in the HER. The reduced HOMO–LUMO gap, increased softness, and high electrophilicity all point to a highly reactive system that is well-suited for the hydrogen evolution reaction. The ability of these systems to efficiently adsorb and interact with molecular hydrogen is further confirmed by the changes in electronic structure upon adsorption, making these doped surfaces strong potential nano candidates for use in HER catalysis.

More specifically, the enhanced catalytic behavior observed in the engineered Si₅₉ nanoclusters can be understood through a synergistic interplay between Ni doping, substitutional boron incorporation, and Au encapsulation. In this system, Ni acts as the primary catalytic center, providing active sites for hydrogen adsorption through favorable metal–hydrogen interactions. Substitutional boron doping introduces electron-deficient sites within the silicon framework, which promotes charge redistribution and increases the electrophilic character of the system, thereby enhancing interaction with hydrogen intermediates. This effect is reflected in the progressive reduction of the HOMO–LUMO energy gap and the increase in electrophilicity and chemical softness, which collectively facilitate charge transfer during the HER process.

Au encapsulation further modulates the local electronic environment of the Ni active sites through metal–support interactions, leading to additional charge redistribution and stabilization of the nanocluster structure. This interaction can influence the d-electron density of Ni, thereby tuning the strength of hydrogen adsorption. As a result, the combined effect of Ni, B, and Au leads to a coordinated modification of both geometric and electronic structures, which governs the adsorption energetics of hydrogen species.

The role of boron concentration (x = 1–3) is particularly significant in controlling the balance between hydrogen adsorption and desorption. Increasing boron content enhances charge redistribution and strengthens hydrogen binding, as evidenced by the trend in ΔG_H*_ values. However, excessive boron incorporation leads to overly strong adsorption, which may hinder hydrogen desorption. Notably, the B₂-doped configuration provides a more favorable balance between adsorption and desorption compared to other compositions, suggesting the existence of an optimal doping regime.

These observations can be rationalized within a descriptor-based framework, where electronic parameters such as the HOMO–LUMO gap, electrophilicity index, and chemical softness serve as key indicators of catalytic activity. Lower energy gaps and higher electrophilicity/softness correspond to enhanced charge transfer capability and stronger interaction with hydrogen intermediates, thereby directly influencing ΔG_H*_. Collectively, this establishes a clear structure–modification–electronic property–catalytic performance relationship, providing mechanistic insight into the design of doped silicon-based nanoclusters for HER applications. This synergistic mechanism highlights the importance of multi-component design strategies in achieving optimized catalytic performance.

#### Band gap variations

**Figure 3:**
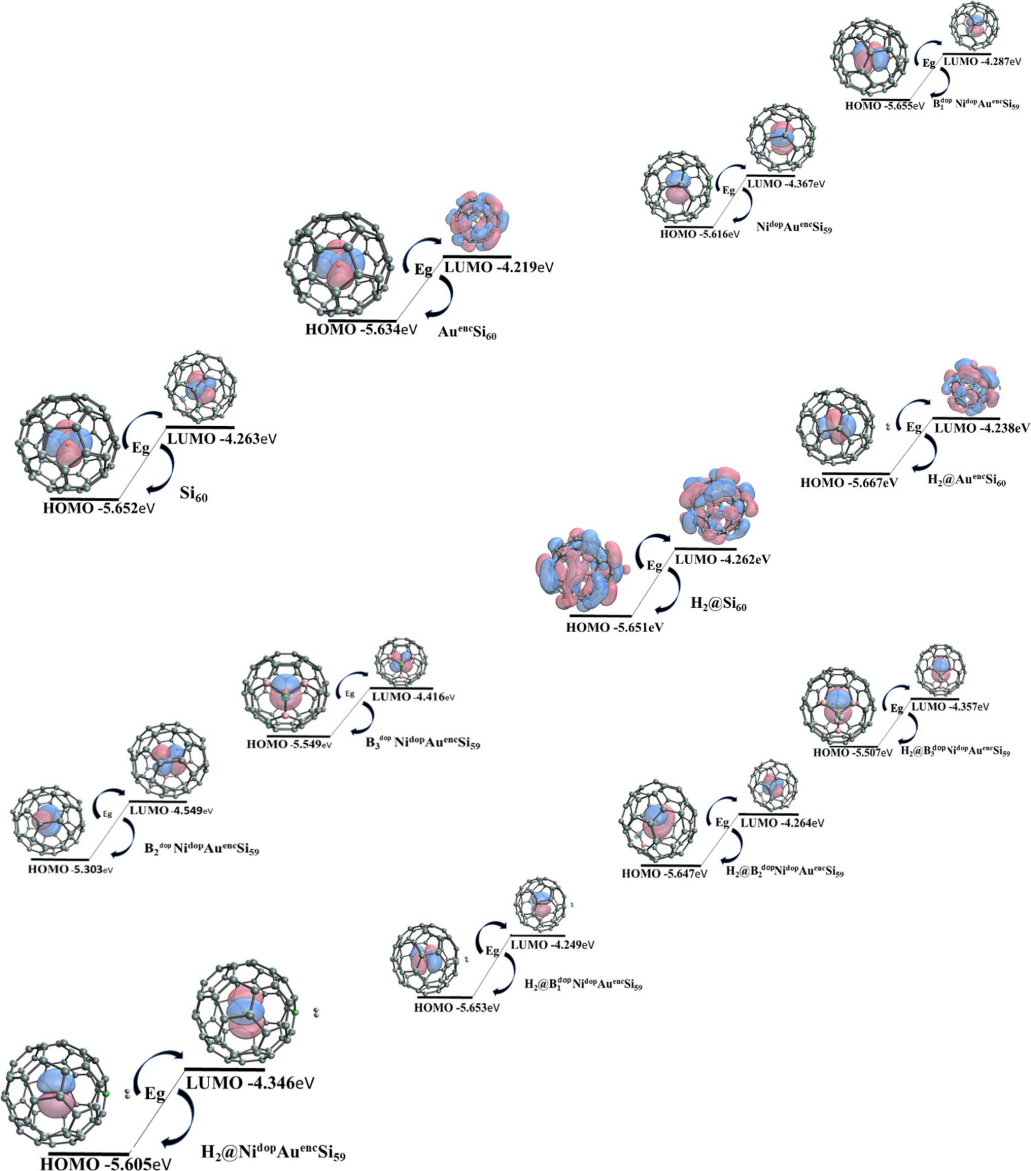
HOMO–LUMO electronic iso-surface mapping of the studied systems, before and post-H_**2**_-adsorption.

The fluctuations of the energy gap (E_g_) across various modeled systems, as shown in Fig. 3 and Table 1, provided valuable insights, particularly concerning hydrogen adsorption and its implications for catalytic activity in the hydrogen evolution reaction (HER), as depicted in Table 1. The energy gap (ΔE_g_), defined as the difference between the highest occupied molecular orbital (HOMO) and the lowest unoccupied molecular orbital (LUMO), is a crucial indicator of a material’s reactivity and stability^40^. Typically, a smaller energy gap indicates higher reactivity and lower stability, characteristics that are beneficial for catalytic applications such as HER, where efficient electron transfer is vital^41^.

The baseline system, Si₆₀, has an energy gap of 1.389 eV as shown in Table 1 and clearly illustrated in Fig. 3. This moderate value indicates a balance between reactivity and stability; while the system possesses some catalytic potential, its relatively higher energy gap suggests that it may not be fully optimized for HER activity. A moderate energy gap allows for some electron mobility, but it may not facilitate the rapid electron transfer required for efficient hydrogen evolution. Thus, when gold (Au) was encapsulated within the Si₆₀ structure (Au^enc^Si_60_), the energy gap slightly increased to 1.414 eV. This increase implies that the system becomes somewhat more stable due to the presence of gold, which can enhance the structural integrity of the material. However, this stabilization may come at the cost of reactivity, as the higher energy gap indicates a reduced likelihood of electron transfer, potentially hindering the processes essential for HER.

Hence, a more pronounced fluctuation occurs with the introduction of nickel into the system (Ni^dop^Au^enc^Si₅₉), where the energy gap decreases to 1.249 eV (see Table 1 and Fig. 3). This reduction is significant as it enhances the system’s reactivity, making it more suitable for HER, as a lower energy gap facilitates easier electron movement between the HOMO and LUMO, promoting faster electron transfer. In catalytic processes like HER, where rapid electron movement is crucial for hydrogen adsorption and reduction, this reduction in the energy gap suggests improved catalytic performance. Nickel doping effectively lowers the energy barrier for electron transfer, which is favorable for hydrogen evolution (see Fig. 3 and Table 1). More so, further modifications, such as the doping of the system with boron, B₁^dop^Ni^dop^Au^enc^Si₅₉, B_2_^dop^Ni^dop^Au^enc^Si₅₉, B_3_^dop^Ni^dop^Au^enc^Si₅₉, reveal additional fluctuations in the energy gap. The most significant reduction occurs in the B₂^dop^Ni^dop^Au^enc^Si₅₉ system, where the energy gap drops to 0.754 eV. This substantial decrease indicates an even higher reactivity compared to the nickel-doped system, as shown in the theoretically calculated value presented in Table 1 and pictorially represented in Fig. 3. This tentatively infers that boron doping significantly enhances the system’s capacity for electron transfer, which is critical for HER. A smaller energy gap implies that the system can more readily facilitate the adsorption and dissociation of molecular hydrogen, a crucial step in the HER process. Thus, the combination of boron and nickel doping creates a synergistic effect, further optimizing the system for catalytic applications.

Upon hydrogen adsorption (H₂@Si₆₀, H₂@Au^enc^Si_60_, H₂@Ni^dop^Au^enc^Si₅₉, etc.), the energy gap values exhibit noticeable changes. For instance, in the case of H₂@Ni^dop^Au^enc^Si₅₉, the energy gap dramatically decreases to 0.629 eV, indicating that hydrogen adsorption significantly enhances the system’s reactivity. This reduction in the energy gap upon hydrogen interaction suggests that the system becomes even more active, facilitating electron transfer during the HER process (see Table 1 and Fig. 3). The ability to adsorb hydrogen while simultaneously lowering the energy gap is a desirable characteristic for any catalytic material involved in HER.

Substantially, the fluctuations in energy gap across the different modeled systems underscore the critical roles of encapsulation and doping in tailoring the electronic properties of Si₆₀-based systems for HER. While the pure silicon system shows moderate potential, nickel doping and boron doping significantly reduce the energy gap, enhancing reactivity and catalytic efficiency. The smallest energy gaps, particularly after hydrogen adsorption indicate that these systems are well-optimized for promoting the electron transfer necessary for HER, positioning them as strong candidates for further exploration in hydrogen evolution catalysis.

### Impact of structure on hydrogen evolution reaction activity

#### NBO (natural bond orbital) analysis of studied surfaces

Natural Bond Orbital (NBO) analysis is a powerful method for examining electron density delocalization and hyperconjugation effects, particularly in the context of intermolecular and intramolecular interactions^42^. This analytical approach provides insights into the interactions within molecular bonds by evaluating the relationships between occupied (donor) and unoccupied (acceptor) orbitals in the systems under study^43^. NBO analysis focuses on bonding and antibonding interactions by identifying potential interactions between filled Lewis-type natural bonding orbitals (donors) and empty non-lewis orbitals (acceptors). The energies associated with these interactions are estimated using second-order perturbation theory, which highlights that the stabilization of a molecule is significantly influenced by delocalization effects, serving as corrections to the basic Lewis structure.

The second-order perturbation interaction energy, denoted as E^(2)^, quantifies the strength of donor–acceptor interactions. It is calculated using the formula:

1 $$E^{\left( 2 \right)} = q_{i } \frac{{F\left( {i,j} \right)^{2} }}{\varepsilon j - \varepsilon i}$$

where q_i_ represents the occupancy of the donor orbital, and (i,j) refers to the interactions between the donor (Lewis-type) and acceptor (non-Lewis) orbitals. This equation captures the stabilizing effect arising from electron interactions between donors and acceptors.

The supporting information Tables S2 and S1 provide essential insights into the behavior of silicon-based nanoclusters before and after hydrogen interaction, offering a framework for understanding their catalytic activity in the hydrogen evolution reaction (HER). By analyzing NBO data and second-order perturbation energy calculations, it was observed that structural and electronic changes influence the surfaces’ ability to effectively catalyze HER. Before hydrogen adsorption, the studied non-hydrogenated systems, Si₆₀, Au^enc^Si_60_, and Ni^dop^Au^enc^Si₅₉ predominantly exhibit SP hybridization, with S-character and P-character values remaining stable at approximately 29% and 71%, respectively. This stability indicates that, despite modifications from metal doping or encapsulation, the structural integrity of these nanoclusters is maintained before hydrogen interaction. Such stability is crucial for HER, as it allows for effective hydrogen atom adsorption, a vital initial step in the reaction.

Hence, upon hydrogen adsorption, significant changes in hybridization patterns were observed, particularly in systems like H₂@Ni^dop^Au^enc^Si₅₉ and H₂@B_x_^dop^Ni^dop^Au^enc^Si₅₉ (where B_x_ represents different boron doping levels). The emergence of SP^2^ and SP^3^ hybridization suggests substantial rehybridization of the orbitals, indicating that the surfaces undergo structural adjustments to accommodate hydrogen. This rehybridization reflects a modification in electronic distribution, enhancing sigma (σ) and pi (π) bonding contributions.

These changes are critical for HER, as they strengthen the interaction between the surface and hydrogen, facilitating improved electron transfer. This is because enhanced electron transfer is essential for driving the hydrogen evolution process, as it reduces the activation energy barrier for hydrogen dissociation, thereby promoting the overall efficiency of the reaction^44^.

The analysis indicates that minor fluctuations in S- and P-Characters following hydrogen adsorption led to significant changes in bond strength and nature, which enhance the surface’s capacity for hydrogen bonding. An increase in P-character is associated with stronger π-bonding, stabilizing adsorbed hydrogen, while an increase in S-character signifies stronger σ-bonding, indicating robust interactions between hydrogen atoms and the surfaces studied. These bonding alterations are critical for the efficiency of the hydrogen evolution reaction (HER), influencing the processes of adsorption, activation, and evolution of hydrogen atoms.

The second table, table S1, presents second-order perturbation energies, which quantify the strength of donor–acceptor interactions between orbitals. Prior to hydrogen adsorption, systems like Si₆₀ and Ni^dop^Au^enc^Si₅₉ exhibit moderate perturbation energy values, reflecting stable interactions that are not excessively strong. This stability is vital for effective hydrogen adsorption and reaction. However, after H_2_ adsorption, perturbation energies rise significantly, especially in doped surfaces such as H₂@Ni^dop^Au^enc^Si₅₉, where the energy reaches 76.68 kJ/mol. This increase indicates strong interactions between adsorbed hydrogen and surface atoms, facilitating efficient HER by lowering the energy barrier for hydrogen dissociation.

Charge transfer delocalization (ΔE) values, which represent the energy difference between donor and acceptor orbitals, also provide valuable insights. Lower ΔE values after hydrogen adsorption suggest enhanced electron transfer capabilities, essential for HER. The reduction in ΔE values reinforces the idea that hydrogen interactions improve electron flow, thereby decreasing the activation energy required for hydrogen dissociation.

Painstakingly, the findings suggest that transition metal-doped and doped systems, particularly those involving Ni and B, are effective catalysts for HER. The combination of increased perturbation energies, decreased ΔE values, and orbital rehybridization indicates that these surfaces are well-suited for hydrogen adsorption and evolution. The structural modifications from doping not only stabilize the surfaces but also enhance their catalytic activity, making them promising candidates for efficient HER applications. The interplay between natural bond orbital (NBO) analysis and perturbation energy calculations underscores the relationship between electronic and structural changes in improving hydrogen interactions, which is crucial for enhancing HER performance. In this context, charge transfer (QCT) is defined as;

2 $${\mathrm{Q}}_{{{\mathrm{CT}}}} = n\left( {\frac{{F_{i,j} }}{{E_{i} - E_{j} }}} \right)^{2}$$

where n represents orbital occupancies, F_**i,j​**_ refers to the off-diagonal elements of the natural bond orbital Fock matrix, and E_i_​ − E_j_​ indicates the diagonal element parameters. The analyzed hydrogenated system-H₂@Si₆₀, H₂@Au^enc^Si_60_, H₂@Ni^dop^Au^enc^Si₅₉, and H₂@B_x_^dop^Ni^dop^Au^enc^Si₅₉ (x = 1, 2, 3)—revealed charge transfer values of 1.75e, 1.089e, 0.422e, 2.283e, 1.134e, and 10.37e, respectively. As such, the significantly high charge transfer value of 10.37e for H₂@B_x_^dop^Ni^dop^Au^enc^Si₅₉ indicates its superior catalytic potential for the hydrogen evolution reaction (HER), suggesting effective electron transfer and enhanced hydrogen dissociation upon boron-doping and hydrogenation.

### Quantum theory of atoms in molecules (QTAIM) analysis

**Figure 4:**
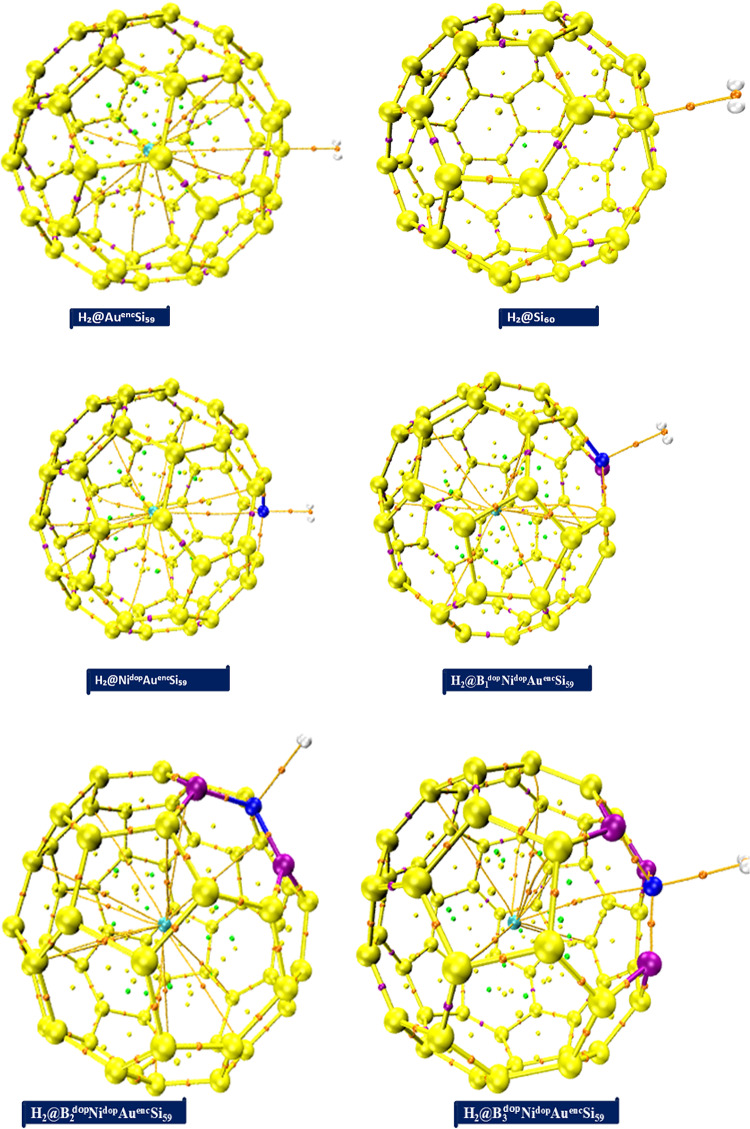
QTAIM Molecular Graph of Interactions in Hydrogen-Adsorbed Systems Studied.

The QTAIM analysis illustrated in Fig. 4 provides valuable insights into the electronic structure and bonding characteristics of hydrogen across various proposed catalyst systems: H₂@Si₆₀, H₂@Au^enc^Si_60_, H₂@Ni^dop^Au^enc^Si₅₉, and the boron-doped variant, H₂@B₂^dop^Ni^dop^Au^enc^Si₅₉. This analysis compares key parameters such as electron density (ρ(r)), Laplacian (∇^2^ρ(r)), kinetic energy (G(r)), potential energy (V(r)), energy density (H(r)), electron localization function (ELF), ellipticity (ϵ), and Hessian eigenvalues (λ). These metrics enhance our understanding of the strength, stability, and nature of the interactions between hydrogen and the catalyst surface, which are critical for optimizing HER efficiency^45^.

Herein, electron density (ρ(r)), which indicates electron concentration at the bond critical point (BCP) was critically paid attention to. As such, we evaluated the bond strength between hydrogen and the proposed catalyst systems. The H₂@Si₆₀ system displays a moderate electron density of 0.213 a.u., suggesting weak hydrogen adsorption primarily through van der Waals forces. Similarly, H₂@Au^enc^Si_60_, with an electron density of 0.211 a.u., shows comparable weak interactions, indicating that gold encapsulation does not significantly enhance hydrogen adsorption. These findings suggest that both systems are suboptimal for HER, where effective hydrogen adsorption and activation are essential.

In contrast, the Ni-doped system, H₂@Ni^dop^Au^enc^Si₅₉, exhibits a slightly higher electron density of 0.217 a.u., indicating that Ni doping may enhance hydrogen adsorption and potentially improve catalytic efficiency. Thus, the boron- doped system, H₂@B₂^dop^Ni^dop^Au^enc^Si₅₉, shows a significantly higher electron density of 0.804 a.u. at the Ni…Si bond as depicted in supporting information Table S3, reflecting a much stronger interaction. This increased electron density is favorable for HER, suggesting enhanced hydrogen adsorption and activation, which are crucial for improved catalytic performance. More so, the Laplacian of the electron density (∇^2^ρ(r)), which assesses electron density distribution, further elucidates bond characteristics. Positive Laplacian values indicate closed-shell interactions typical of weak bonding, such as van der Waals or ionic interactions^43^. Hence, both H₂@Si₆₀ and H₂@Au^enc^Si_60_ exhibit similar positive Laplacian values (0.619 a.u.), reinforcing the notion of weak, non-covalent interactions with hydrogen. However, the Laplacian increases slightly in the Ni-doped system (0.636 a.u.), suggesting a transition toward stronger, more localized interactions. The highest Laplacian value of 0.759 a.u. in H₂@B₂^dop^Ni^dop^Au^enc^Si₅₉ indicates a significant enhancement in bonding interactions due to boron doping, further supporting the potential of this system for HER applications. This stronger electron localization at the bond critical points suggests that the B₂^dop^Ni^dop^Au^enc^Si₅₉ system may facilitate improved hydrogen adsorption, a critical factor for efficient HER catalysis.

Crucially, as also shown in Table S3, the kinetic energy G(r) and potential energy V(r) at the bond critical point provide critical insights into bond strength and electron confinement. Higher kinetic energy values indicate stronger electron confinement, while more negative potential energy values suggest stronger attractive forces within the bond^46^. The Ni-doped system, H_2_​@Ni^dop^Au^enc^Si_5​9_, shows a significantly higher kinetic energy of 0.183 a.u. compared to H_2_​@Si_5​9_ and H_2_​@Au^enc^Si_5​9_ (both approximately 0.152 a.u.), indicating enhanced electron confinement and stronger hydrogen bonding. Additionally, the potential energy in the Ni-doped system is more negative (− 0.207 a.u.), reinforcing the conclusion that the Ni…H interaction is stronger than in the other systems. This stronger bonding interaction promotes hydrogen adsorption and activation, which are essential for the hydrogen evolution reaction (HER). Substantially, the boron- doped system, H_2_​@B_2_​^dop^Ni^dop^Au^enc^Si_5​9_, exhibits an even more negative potential energy of − 0.786 a.u., highlighting the significantly enhanced hydrogen bonding interaction due to boron doping. This suggests that boron plays a crucial role in increasing the catalytic activity of the Ni-doped system by strengthening hydrogen adsorption, making it a particularly promising catalyst for HER.

Energy density H(r), which combines G(r) and V(r), provides insight into bond nature, distinguishing covalent from non-covalent interactions. For H_2_​@Si_60_ and H_2_​@Au^enc^Si_60_, slightly negative H(r) values (− 0.150 a.u.) indicate weak covalent interactions, limiting their hydrogen evolution reaction (HER) effectiveness. In contrast, H_2_​@Ni^dop^Au^enc^Si_59_ shows a more negative H(r) (− 0.240 a.u.), suggesting stronger covalent interactions favorable for hydrogen adsorption. The highly negative H(r) of − 0.789 a.u. for H_2​_@B_2_​^dop^Ni^dop^Au^enc^Si_59_​indicates very strong covalent bonds, making it particularly suited for HER. The electron localization function (ELF) and ellipticity ϵ further clarify bond stability. ELF values around 0.846 in H_2_​@Si_59_ and H_2_​@Au^enc^Si_60_ suggest weak interactions, while a decrease to 0.654 in the Ni-doped system indicates more delocalized electrons, aiding electron transfer during HER. The ELF drops to 0.438 in the boron- doped system, enhancing electron mobility for efficient hydrogen reduction. More so, ellipticity, ϵ indicates bond stability; low values in H_2_​@Si_59_ and H_2_​@Au^enc^Si_60_ (~ 0.299) suggest stable but weak bonds. Higher ellipticity in H_2_​@Ni^dop^Au^enc^Si_59_ (0.468) indicates stronger, more easily distorted bonds, while the very low ellipticity (0.039) in H_2​_@B_2_​^dop^Ni^dop^Au^enc^Si_59_ reflects highly stable bonds, suggesting effective HER due to strong hydrogen-catalyst interactions.

Building on these crucial kingpins, the analysis of bond critical points (BCPs) and bonding energies (BE) also reveals that Ni-doped systems (EB₆₂–Ni₁₆₁ and EB₆₃–Ni₁₆₁) exhibit similar binding energies (~ − 188 to − 189 kcal/mol), indicating strong interactions and stability. The electron density at BCPs (0.216–0.213 a.u.) suggests reasonably strong hydrogen adsorption, though not optimal for HER. Both systems show similar Laplacian values (∇2ρ(r) ~ 0.751–0.746 a.u.), indicating moderately closed-shell interactions. The potential and kinetic energies at BCPs are also comparable, reflecting similar bonding characteristics.

The energy density (H(r) ~ − 0.780 to − 0.777 a.u.) indicates covalent interaction components essential for efficient hydrogen activation. The low ellipticity (0.023–0.022) and Hessian eigenvalue (~ 0.977–0.978) further confirm bond stability, suggesting that Ni-doping enhances bond strength and stability, potentially improving HER activity. In contrast, the boron- doped system shows weaker bonding (BE: − 179 kcal/mol for EN₁₆₀–H₅₇ and − 163 kcal/mol for EN₁₆₀–B₆₁) but maintains moderate electron density (0.807 a.u. for EN₁₆₀–H₅₇ and 0.735 a.u. for EN₁₆₀–B₆₁), indicating significant hydrogen adsorption. The Laplacian values differ from the Ni-doped systems, with EN₁₆₀–H₅₇ showing a negative value (− 0.106 a.u.), suggesting more covalent character, which is favorable for HER. In contrast, EN₁₆₀–B₆₁ has a positive Laplacian (0.173 a.u.), indicating a weaker interaction. Overall, these findings highlight the impact of structural modifications on hydrogen bonding and catalytic efficiency in HER.

For EN₁₆₀–H₅₇, G(r) is higher at 0.408 a.u., and the corresponding V(r) is less negative (− 0.531 a.u.), suggesting stronger electron localization and bond strength compared to EN₁₆₀–B₆₁, which shows lower kinetic energy (0.154 a.u.) and less negative potential energy (− 0.739 a.u.). This is consistent with the expectation that the boron- doped system introduces stronger interactions with hydrogen, particularly through the EN₁₆₀–H₅₇ bond, which may facilitate better hydrogen activation for HER. The energy density H(r) for the boron- doped system is less negative than that in the Ni-doped systems (− 0.231 a.u. for EN₁₆₀–H₅₇ and − 0.791 a.u. for EN₁₆₀–B₆₁), reflecting the more balanced nature of attractive and repulsive forces in these bonds. However, the ellipticity ϵ is significantly lower for the EN₁₆₀–H₅₇ bond (0.149), and even lower for EN₁₆₀–B₆₁ (0.027), suggesting that these bonds are more stable and less prone to distortion than the bonds in the Ni-doped systems. This enhanced stability could be a beneficial trait for HER catalysis, where bond stability and efficient hydrogen activation are critical. Finally, the Hessian eigenvalues of 0.768 for EN₁₆₀–H₅₇ and 1.026 for EN₁₆₀–B₆₁ confirm that the boron- doped system is overall more stable and more capable of maintaining its structural integrity during catalysis^38^. This structural stability, combined with the strong hydrogen bonding interactions, points to the potential for the boron- doped Ni-doped system to serve as an efficient catalyst for the hydrogen evolution reaction, with promising implications for enhancing HER efficiency.

Thus, from the garnered data analyzed herein, Ni-doped systems exhibited improved hydrogen bonding and stability over undoped systems. However, the addition of boron doping further enhances these properties. The boron-doped system shows stronger and more stable hydrogen interactions, indicating the potential for superior performance in hydrogen evolution reactions (HER). Key factors include increased electron density, greater covalent character, and enhanced bond stability, positioning it as a promising candidate for catalytic hydrogen evolution processes.

### Density of state (DOS), Charge Transfer Analysis: Correlating Electronic Structure with HER Activity

**Figure 5:**
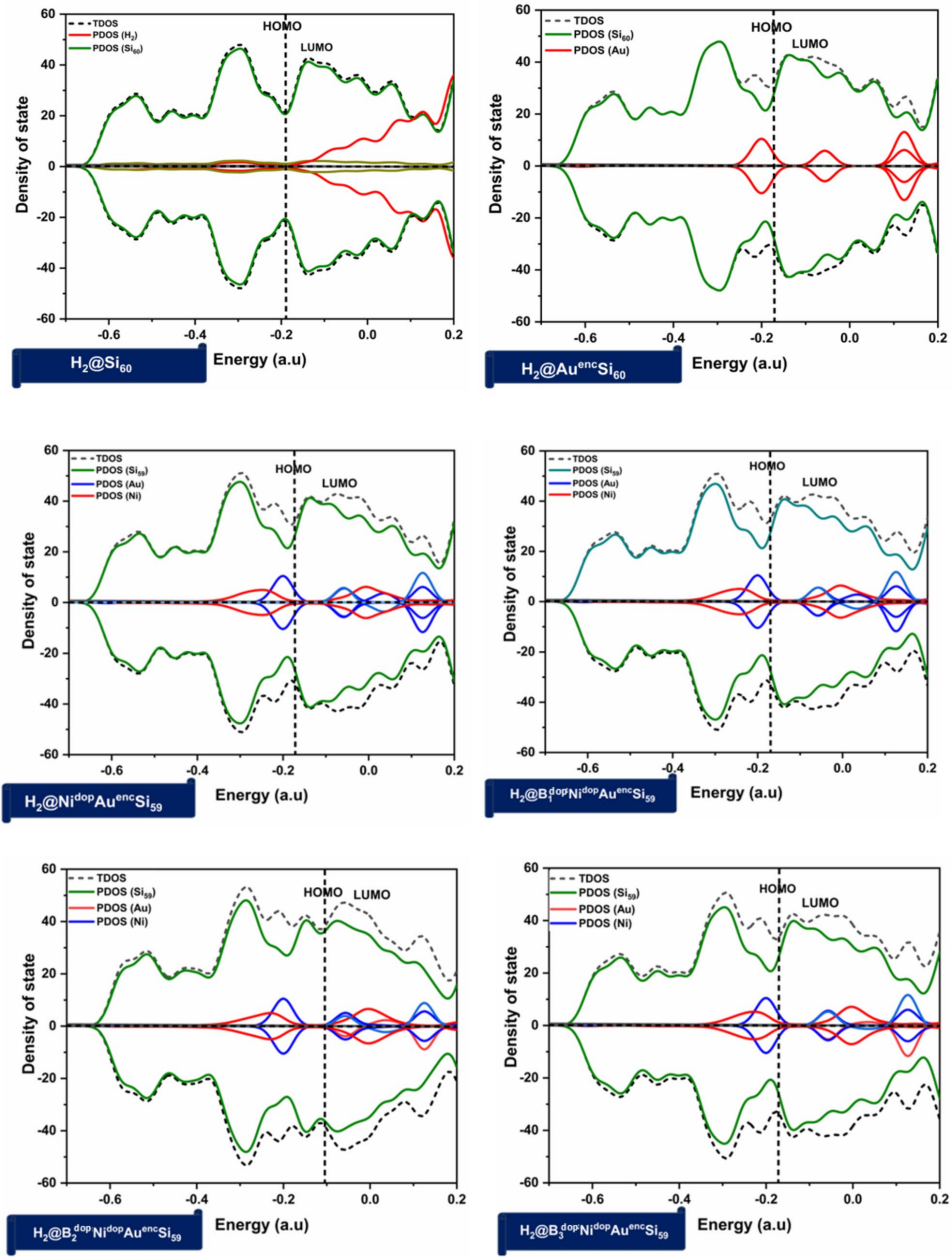
Density of States (DOS) Plots Showing Atomic Contributions to Occupied and Unoccupied Electronic States.

The density of states (DOS) plots for the hydrogen-adsorbed systems provide valuable insights into their electronic structures and catalytic potential, particularly for the hydrogen evolution reaction (HER). As illustrated in Fig. 5, the total DOS (TDOS) and partial DOS (PDOS) reveal how atomic constituents—H₂, Si₆₀, Ni, Au, and B—contribute to electronic states near the Fermi level, where reactivity is most pronounced. These electronic states are directly tied to parameters critical for HER, such as charge transfer, adsorption strength, and orbital hybridization. For the H₂@Si₆₀ system, the PDOS reveals notable activity around the HOMO (− 0.6 to − 0.2 a.u) and LUMO (− 0.1 to 0.1 a.u) regions, with the Si₆₀ framework dominating these contributions. However, the minimal PDOS involvement of H₂ suggests weak orbital overlap and negligible interaction between hydrogen and the cluster. This aligns with the low QCT value of 1.75e, reflecting limited charge transfer, and a relatively moderate HOMO–LUMO gap (~ 0.205 a.u), indicative of restricted electronic mobility and modest HER catalytic capacity.

The H₂@Au^enc^Si₆₀ system introduces gold, modifying the electronic structure near the Fermi level. Strong PDOS peaks attributable to Au are observed between − 0.3 and 0.2 a.u, reducing the HOMO–LUMO gap (~ 0.196 a.u). While this suggests enhanced electron accessibility, the charge transfer remains moderate at 1.089e, consistent with weak interaction between Au and H₂. Despite the enriched electronic states, the catalytic behavior remains suboptimal without stronger orbital hybridization or enhanced donor–acceptor interactions. Upon Ni doping, the H₂@Ni^dop^Au^enc^Si₅₉ system shows increased PDOS activity near the LUMO (− 0.3 to − 0.2 a.u), suggesting that Ni introduces accessible acceptor states favorable for electron transfer. This is corroborated by a rise in perturbation energy (up to 76.68 kJ/mol) and a moderate QCT value of 0.422e, indicating that Ni enhances donor–acceptor interactions at the adsorption site. The narrowing of the HOMO–LUMO gap (~ 0.195 a.u) supports a more conductive electronic profile, yet the overall HER enhancement is limited without additional charge delocalization pathways.

The H₂@B_x_^dop^Ni^dop^Au^enc^Si₅₉ systems (x = 1, 2, 3) reveal the most significant electronic and catalytic transformation. In particular, B₃ doping enhances PDOS near the Fermi level with notable contributions from B, Ni, and Au. This coincides with the highest QCT value of 10.37e, demonstrating extensive charge transfer from the surface to the adsorbed H₂. The second-order perturbation analysis further confirms robust donor–acceptor interactions and rehybridization phenomena (SP^2^/SP^3^), which facilitate efficient σ and π bonding between the cluster and hydrogen. Incremental boron doping (B₁ → B₂ → B₃) shows a systematic enhancement in PDOS intensity near the Fermi level, narrowing the HOMO–LUMO gap (~ 0.193 to ~ 0.198 a.u) and improving orbital overlap. The rising QCT values (2.283e and 1.134e for B₁ and B₂, respectively) confirm that electron delocalization strengthens with increased Boron content. These trends reflect improved electronic communication between active sites and adsorbed H₂, enhancing HER kinetics by reducing the energy barrier for hydrogen dissociation and facilitating electron transfer.

Additionally, in our initial PDOS analysis (Fig. 5), the increased density of states near the Fermi level observed in the B₃- doped systems is primarily attributed to the hybridization interactions among B, Ni, and Au orbitals. Although the specific contribution of B atoms was not explicitly delineated in the original figure, the role of B in modifying the electronic structure is significant. The B atoms interact with the Ni and Au atoms, leading to the formation of new electronic states that enhance the PDOS at energies close to the Fermi level, thereby facilitating improved electron transfer during HER. To illustrate this more clearly, we have included additional PDOS plots focusing solely on the B atom contributions (see Supplementary Material). These plots confirm that B orbitals significantly contribute to the states near the Fermi level, supporting our interpretation that B doping plays a crucial role in tuning the electronic properties and catalytic activity of the nanostructures.

In summary, while HOMO–LUMO gap reductions point to general electronic flexibility, the localized PDOS contributions from catalytically active centers (Ni, B, Au) and their correlation with NBO-derived QCT values and perturbation energies provide a deeper understanding of HER reactivity. The enhanced DOS near the Fermi level, especially in B, Ni-doped systems, combined with high charge transfer and orbital rehybridization, establishes a compelling electronic framework for efficient hydrogen evolution catalysis.

### Hydrogen evolution reaction (HER) protocol

The hydrogen evolution reaction (HER) occurs at the cathode of an electrolytic cell, with variations in mechanism between acidic and alkaline environments due to different proton sources^47–53^. In acidic media, the HER begins with the **Volmer step**, or the electrochemical adsorption step, where H + from the solution accepts an electron and binds to the catalyst surface, forming a hydrogen intermediate H^∗^. This step is essential for subsequent hydrogen gas H_2_ production. In our study, we are particularly focused on the **Heyrovsky step** in an acidic environment, as this is the most feasible pathway for H_2_ evolution in our setup. Here, the H^∗^ intermediate on the surface combines with an additional H^+^ from the solution and an electron, producing H_2_, as shown in Fig. 5. In our case, we have adsorbed H_2_ directly onto the catalytic surface rather than H^+^, limiting the process to the Heyrovsky mechanism through **heterolytic cleavage**, depicted in Fig. 5. The heterolytic cleavage of H_2_ generates H^∗^, enabling progression through the Heyrovsky pathway.

**Figure 6:**
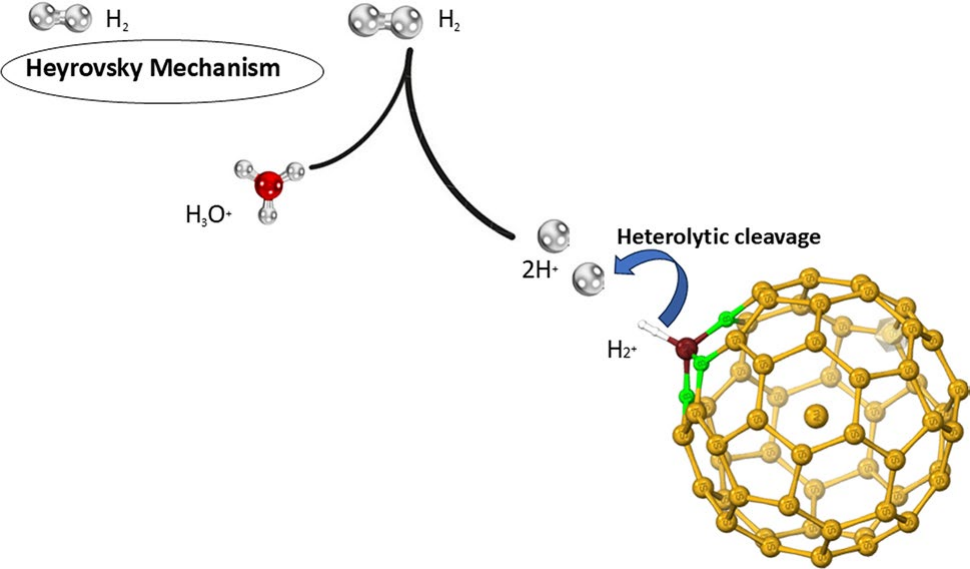
Example Volmer-Heyrovsky Mechanisms for the studied electrocatalytic HER Silicon-based systems**.**

In an alkaline medium, water splitting broken down into three elementary steps provides the necessary H^∗^ intermediate. This leads to a higher energy requirement to produce H^∗^, slowing the HER kinetics in alkaline conditions. Using gold (Au) as a reference, HER rates are often two to three orders of magnitude slower in alkaline media than in acidic media due to the higher energy barrier in water splitting. To understand the effects of **encapsulation and doping** on an all-silicon nanostructure under study, we meticulously examine the transition state complexes, considering three states: an initial state (H^+^ + e −), an intermediate adsorbed configuration (H^∗^), and the final product H_2_ ​ (H^∗^  + H^+^  → H_2_). In most cases, the Gibbs free energy of the intermediate state serves as an effective indicator of HER activity. The energy profile for the Volmer and Heyrovsky mechanisms for the engineered nanostructures is illustrated in Figs. 6 and 8. In the initial state, a physiosorbed proton binds to the doped metal site at a distance of 2.414 Å. During the transition state, the H-bond elongates to 2.814 Å before desorption from the Si–H bond, forming a Ni–H bond at 2.390 Å. The free energy of this reaction is calculated to be − 1.80 eV, with an associated energy barrier of 150 kJ/mol (or 1.55 eV), indicating an exothermic reaction. This exothermic nature is evident from Fig. 8, where the reactants begin at approximately − 150 kJ/mol, and the products end at a lower energy level, close to − 180 kJ/mol, suggesting an energy release of around 30 kJ/mol. Therefore, this pathway demonstrates that the **Volmer-Heyrovsky route** is favorable. In the Heyrovsky step specifically, the chemisorbed proton and the transition-state proton approach to form molecular hydrogen^47^. Here, the H_2_​ bond length is about 0.372 Å shorter than in the initially adsorbed H_2_​ molecule (0.743 Å) or the transition-state structure (2.814 Å), underscoring the efficiency of the Heyrovsky mechanism on our catalytic system. Table 2 shows how the bond length of hydrogen molecules slightly undergoes expansion upon adsorption onto the modeled electrocatalytic silicon-based surfaces from 0.743 Å to 0.8096 Å and 0.797 Å, being the highest observed so far. This could suggest that there’s a huge possibility of the H–H bond splitting into two protons 2H^+^ hence following the Heyrovsky mechanism or reaction pathway as shown in Fig. 5.

**Table 2:** Showing bond length of H_2_ molecule before and post adsorption.

Systems	Before adsorption (Å)	Post adsorption (expansion) (Å)
H_2_	0.743	–
H_2_@Si_60_	0.743	0.7435
H_2_@Au^enc^Si_60_	0.743	0.7452
H_2_@Ni^dop^Au^enc^Si_59_	0.743	0.7972
H_2_@B_1_^dop^Ni^dop^Au^enc^Si_59_	0.743	0.8096
H_2_@B_2_^dop^Ni^dop^Au^enc^Si_59_	0.743	0.7968
H_2_@B_3_^dop^Ni^dop^Au^enc^Si_59_	0.743	0.7682

Hence, the hydrogen evolution reaction (HER) typically occurs through two or three steps, depending on the mechanistic pathway, either the Volmer–Heyrovsky or the Volmer–Tafel mechanism, as stated earlier. The initial step generally involves the adsorption of hydrogen atoms (H*) onto the catalyst surface:

#### Volmer step (adsorption)

H^+^ + e^−^ + * →H^∗^ ; Here, a proton and an electron combine and adsorb onto the catalyst, forming an adsorbed hydrogen atom H*. Subsequently, the process proceeds through either;

#### Heyrovsky step (electrochemical desorption)

H^∗^ + H^+^ + e^−^ → H_**2​**_ + ^∗^; In this step, an adsorbed H* interacts with a proton and an electron to produce molecular hydrogen (H₂), which then desorbs from the surface.

#### Tafel step (chemical desorption)

2H^*^ → H_**2**_​ + 2^*^; Here, two adsorbed hydrogen atoms combine to form H₂, which leaves the surface, completing the reaction.

Hence, the hydrogen adsorption free energy (ΔG_H_) is a critical descriptor for evaluating the catalytic performance of materials toward the hydrogen evolution reaction (HER). An optimal HER catalyst must strike a balance in hydrogen binding: if ΔG_H_* is too positive, hydrogen adsorption becomes unfavorable, leading to insufficient surface coverage of H* intermediates. Conversely, overly negative ΔG_H_* values indicate strong hydrogen binding, which impedes the desorption step and hinders the release of H₂ gas. Therefore, a ΔG_H_* value close to 0 eV is generally considered ideal, as it reflects a thermodynamically balanced interaction that facilitates both adsorption and desorption steps essential for efficient HER activity.

In this study, a series of Si_59_-based optimized nanostructures were investigated, including Au- and Ni-modified variants, to evaluate their HER catalytic potential through computed ΔG_H_ values. Among the tested configurations, the H_2_@B₂^dop^Ni^dop^Au^enc^Si₅₉ structure exhibited the ΔG_H_* value closest to the thermodynamic optimum (–0.836 eV). This result suggests a more balanced hydrogen adsorption behavior compared to other candidates such as H₂@Si₆₀ (–0.847 eV) or H₂@B_**3**_^dop^Ni^dop^Au^enc^Si₅₉ (–0.842 eV), which demonstrate stronger binding and thus less favorable desorption characteristics. Although none of the studied materials match the benchmark ΔG_H_* of Pt(111) (~ –0.09 eV)^45^, the relative proximity of the B_2_- doped system to the optimal range highlights its potential as a promising HER electrocatalyst for further modification or experimental validation.

### Adsorption Dynamics and Electrocatalysis in Hydrogen Evolution

In heterogeneous catalysis, adsorption plays a crucial role by allowing reactants to attach to the catalyst’s surface, thereby facilitating the transformation into products^54^. In this context, the adsorbate refers to the reacting molecules, while the solid catalyst surface functions as the adsorbent. The effectiveness of adsorption is influenced by the energy barrier to dissociation. Surfaces that are less thermodynamically stable are often more reactive and are therefore preferred for investigating surface reactivity.

This study evaluates the electrocatalytic behavior of pristine and engineered silicon-based nanoclusters, Si₆₀, Au^enc^Si₆₀, Ni^dop^Au^enc^Si₅₉, and the boron- doped variants B₁^dop^Ni^dop^Au^enc^Si₅₉, B₂^dop^Ni^dop^Au^enc^Si₅₉, and B₃^dop^Ni^dop^Au^enc^Si₅₉—with respect to the hydrogen evolution reaction (HER). Table 3 summarizes the computed adsorption energies for molecular and atomic hydrogen on these surfaces. Notably, engineered nanoclusters such as H₂@Ni^dop^Au^enc^Si₅₉ exhibit strong binding with molecular hydrogen, with adsorption energies of –0.6026 eV and –0.6079 eV, indicative of chemisorption. Thus, the adsorption energy for molecular hydrogen (H₂) was computed using the following equation:

3 $$E_{ads}^{H2} = \, E_{(Total)} - \, E_{(Surface)} {-}\frac{1}{2 } E_{(H2)}$$

**Table 3:** Key adsorption parameters essential for catalytic activity, including adsorption energy ($\Delta E_{ads}^{H}$), and Gibbs free energy ($\Delta G_{ads}^{H}$) were evaluated for the engineered Si_60_ surfaces studied.

Models/Surfaces	(ΔE^H^_ads_) eV	(ΔG^H^_ads_) eV
H_2_@Si_60_	− 0.5873	− 0.847
H_2_@Au^enc^Si_60_	− 0.5967	− 0.837
H_2_@Ni^dop^Au^enc^Si_59_	− 0.6026	− 0.837
H_2_@B_1_^dop^Ni^dop^Au^enc^Si_59_	− 0.6010	− 0.841
H_2_@B_2_^dop^Ni^dop^Au^enc^Si_59_	− 0.5977	− 0.836
H_2_@B_3_^dop^Ni^dop^Au^enc^Si_59_	− 0.6079	− 0.842

In this equation, $E_{ads}^{H2}$ represents the adsorption energy of the hydrogen molecule, E_***Total***_​ is the total energy of the system after adsorption, E_***Surface***_​ is the energy of the bare surface, and E_**(H2​)**_ is the energy of an optimized hydrogen molecule in the gas phase. The corresponding Gibbs free energies of adsorption (ΔG_H_) for H₂@Ni^dop^Au^enc^Si₅₉ and H₂@B₃^dop^Ni^dop^Au^enc^Si₅₉ are –0.837 eV and –0.842 eV, respectively. While these negative ΔG_H_ values confirm the spontaneity of hydrogen adsorption, they deviate from the optimal ΔG_H_ ≈ 0 eV typically associated with efficient HER catalysts. According to electrocatalytic principles, a ΔG_H_ value significantly below zero suggests overly strong binding of hydrogen intermediates, which may hinder their desorption and thus impede catalytic turnover. Therefore, these systems, despite exhibiting spontaneous adsorption, may face limitations in the later stages of HER (Heyrovsky or Tafel steps). Nonetheless, their strong binding behavior implies a potentially favorable Volmer step, making them promising candidates for further modification. Future strategies could involve fine-tuning the electronic properties or introducing co-catalysts to shift ΔG_H_ closer to the thermodynamic ideal. Moderate adsorption behavior is also observed for atomic hydrogen on surfaces such as H₂@B₁^dop^Ni^dop^Au^enc^Si₅₉, Si₆₀, and H₂@B₂^dop^Ni^dop^Au^enc^Si₅₉. For instance, H₂@Au^enc^Si₆₀ demonstrates an adsorption energy of –0.5967 eV and a ΔG_H_ of –0.837 eV, again suggesting spontaneous chemisorption, but with the same limitations mentioned above. To further contextualize the catalytic performance of the studied systems, it is instructive to compare the calculated hydrogen adsorption free energy (ΔG_H_) with that of benchmark catalysts. Platinum, particularly Pt(111), is widely regarded as the state-of-the-art HER catalyst, exhibiting a near-thermoneutral ΔG_H*_ value of approximately − 0.09 eV, which is considered optimal for HER activity. In comparison, the best-performing system in this study, B₂-doped Ni–Au–Si₅₉, exhibits a ΔG_H*_ value of − 0.836 eV, indicating significantly stronger hydrogen binding relative to Pt. This suggests that hydrogen adsorption on the proposed system is too strong, which may hinder efficient hydrogen desorption during the HER process. Therefore, further tuning of the electronic structure is required to bring the adsorption energy closer to the thermoneutral regime for optimal catalytic performance. Future studies will focus on optimizing dopant concentration and spatial configuration to achieve improved catalytic behavior. In addition to benchmarking against noble metal catalysts, it is also relevant to compare the proposed systems with other non-noble metal HER catalysts, such as transition metal phosphides, carbides, and sulfides, which have demonstrated promising activity and improved conductivity^54–56^. Compared to these materials, silicon-based nanoclusters offer several advantages, including the natural abundance and low cost of silicon, structural tunability through substitutional doping, and the ability to precisely modulate electronic properties at the nanoscale^57^. These features enable systematic optimization of catalytic behavior through controlled compositional design.

However, certain limitations should also be considered. Silicon-based systems generally exhibit lower intrinsic electrical conductivity compared to transition metal carbides or phosphides, which may impact charge transfer during catalysis^53,55^. In addition, surface oxidation of silicon under operating conditions may affect long-term stability, although this can potentially be mitigated through appropriate surface engineering or encapsulation strategies^58^. Despite these challenges, the tunability and design flexibility of doped silicon nanoclusters position them as promising candidates for next-generation HER catalysts.

As illustrated in Fig. 1, bond lengths between molecular hydrogen and the catalyst surfaces offer further insight into adsorption strength. For example, Ni^dop^Au^enc^Si₅₉ and B₁^dop^Ni^dop^Au^enc^Si₅₉ exhibit bond lengths of approximately 2.399 Å, while B₂^dop^Ni^dop^Au^enc^Si₅₉ and B₃^dop^Ni^dop^Au^enc^Si₅₉ show slightly shorter distances of 2.394 Å, indicative of stronger interactions. Similarly, pristine Si₆₀ and Au^enc^Si₆₀ also show bond lengths around 2.394 Å. These structural findings align with the electronic properties of the systems, particularly their HOMO–LUMO energy gaps. For example, H₂@Au^enc^Si₆₀ and H₂@B₁^dop^Ni^dop^Au^enc^Si₅₉ possess energy gaps of 1.429 eV and 1.404 eV, respectively, with HOMO values of –5.667 eV and –5.653 eV. These values correlate with stronger hydrogen adsorption, as reflected by the shorter bond lengths.

### Gibb’s free energy

To assess the catalytic performance of engineered nanostructures for the hydrogen evolution reaction (HER), we conducted a comparative analysis of the Gibbs free energy associated with hydrogen adsorption. The Gibbs free energy for the molecular hydrogen adsorption on the surfaces of the engineered nanoclusters was calculated using the following equation:

4 $$\Delta G_{H} = E_{ads} - \Delta E_{ZPE} - T\Delta S$$

In this equation, $E_{ads}$ represents the enthalpy related to hydrogen chemisorption, $\Delta E_{ZPE}$ accounts for the zero-point energy difference between hydrogen in its gaseous state and as a molecule (H₂), which is typically around 0.04 eV at room temperature. The term ΔS denotes the change in entropy, which was adjusted by a standard correction of − 0.20 eV for the systems under investigation.

For theoretical analyses of HER, a simplified expression for Gibbs free energy is often used:

5 $$\Delta G_{H} = E_{ads} + 0.24\;{\mathrm{eV}}$$

**Figure 7:**
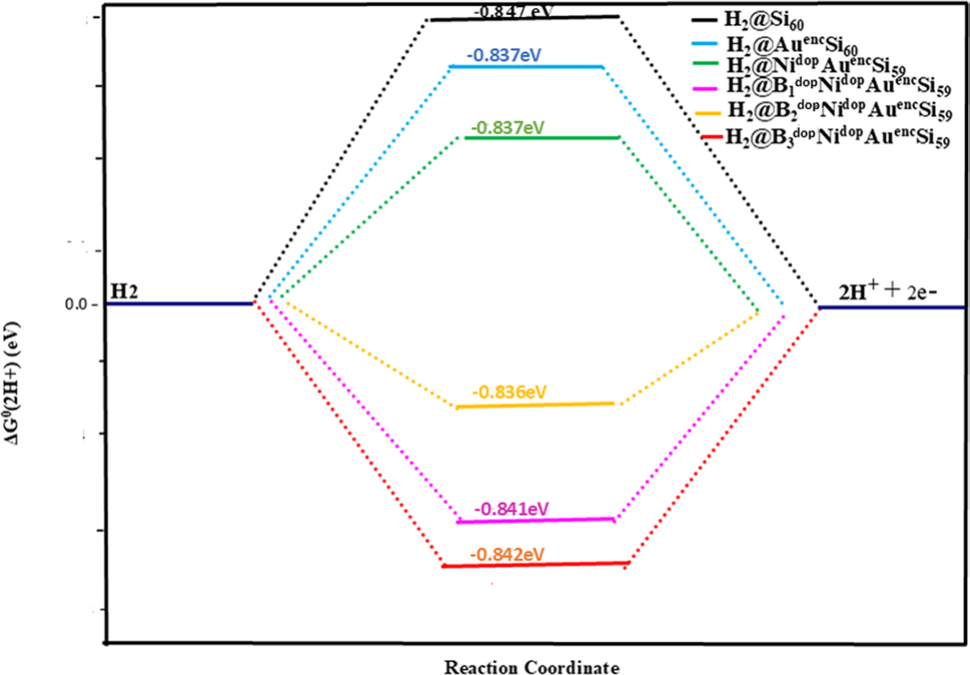
Gibbs Free Energy of Hydrogen Adsorption on Various Engineered Nanostructures: H_2_@Si_60_, H_2_@Au^enc^Si_60_, H_2_@Ni^dop^Au^enc^Si_59,_ H_2_@B_2_^dop^Ni^dop^Au^enc^Si_59,_ H_2_@B_1_^dop^Ni^dop^Au^enc^Si_59,_ and H_2_@B_3_^dop^Ni^dop^Au^enc^Si_59**.**_

According to Sabatier’s principle, optimal catalytic performance in HER requires a balance in the strength of hydrogen adsorption: weak adsorption can impede hydrogen desorption, while excessively strong adsorption can hinder desorption. The computed Gibbs free energies, presented in Fig. 7 and summarized in Table 3, were highly negative for all tested surfaces, which, however, translates to suboptimal, indicating their potential as effective catalytic materials. Hence, among the studied configurations, the H_2_@B_2_^dop^Ni^dop^Au^enc^Si_59_ structure exhibits the ΔG_H__ads value closest to the thermodynamic optimum (− 0.836 eV), suggesting it offers relatively more favorable hydrogen adsorption characteristics and thus better HER catalytic potential compared to the other candidates, while still far from ideal (0 eV), it is the best-performing candidate in this set, as can be seen in Table 3. Notably, H_2_@B_1_^dop^Ni^dop^Au^enc^Si_59_ and H_2_@B_3_^dop^Ni^dop^Au^enc^Si_59_ exhibited more negative free energy values, while H₂@Si_60_ showed a less favorable free energy of − 0.847 eV. Tentatively, H_2_@B_2_^dop^Ni^dop^Au^enc^Si_59_ was optimally positioned according to Sabatier’s guideline, exhibiting a closer to zero $\Delta G$ value, offering a better balance between adsorption and desorption. Based on these findings, we anticipate the efficiency of the studied systems as potential electrocatalysts for HER to follow the order: H_2_@B_2_^dop^Ni^dop^Au^enc^Si_59_ > H_2_@Ni^dop^Au^enc^Si_59_ > H_2_@Au^enc^Si_60_ > H_2_@B_1_^dop^Ni^dop^Au^enc^Si_59_ > H_2_@B_3_^dop^Ni^dop^Au^enc^Si_59_ > H_2_@Si_60_, under conditions of neutral pH and zero internal energy. These results confirm that a systematic approach involving surface doping, and metal encapsulation significantly enhances HER activity, making these engineered doped nanoclusters highly suitable for catalytic applications in hydrogen production. It is important to note that ΔG_H_ serves as a thermodynamic descriptor of HER activity, while kinetic parameters such as overpotential and Tafel slope are not directly accessible from the present computational framework. Accurate evaluation of these metrics would require explicit electrochemical modeling or experimental measurements. Therefore, the present analysis focuses on thermodynamic trends, which are widely accepted as primary indicators of catalytic performance.

### Comparative Analysis of Transition States and Energy Barriers

**Figure 8:**
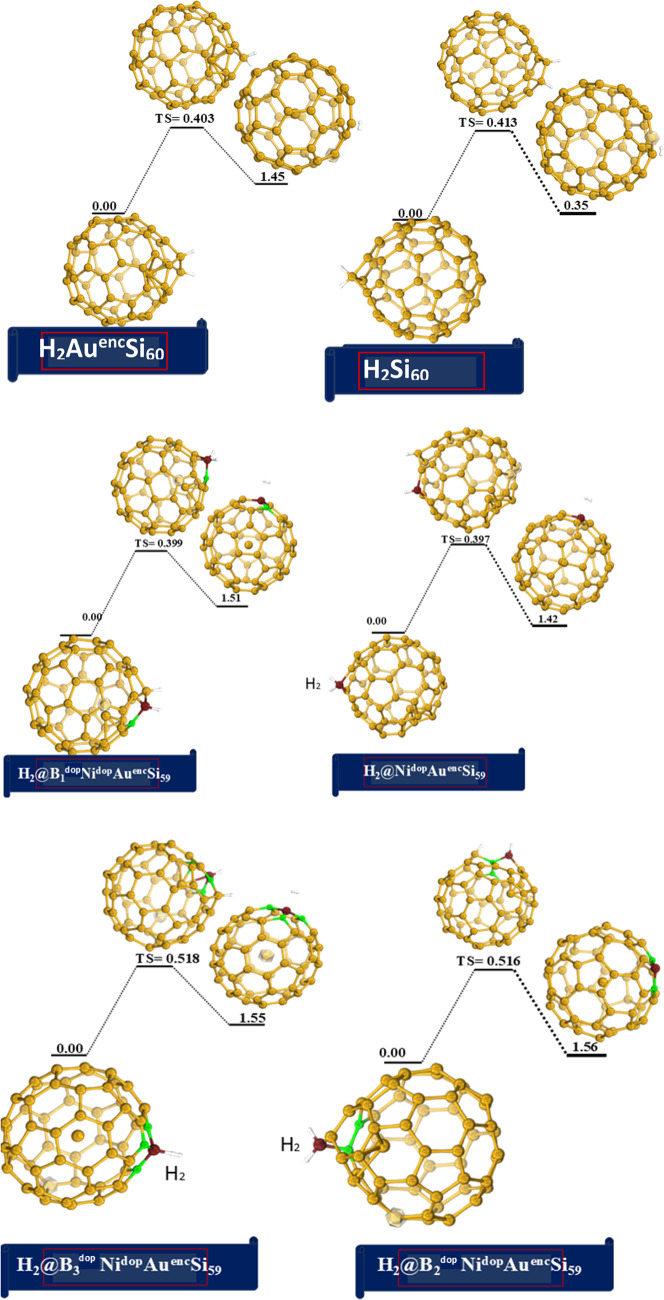
Depiction of Hydrogen Adsorption and H₂ Formation Pathways on H₂@Si₆₀ and Modified Surfaces during HER at pH = 0 and U = 0.

**Figure 9:**
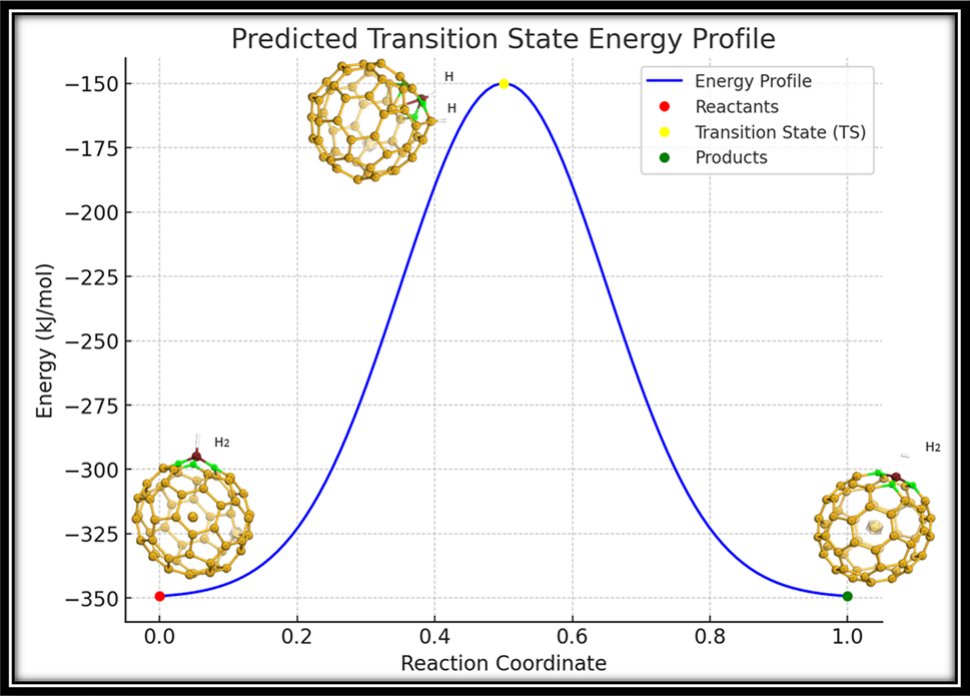
Python-graphical illustration of transition state energy barrier profile for HER activity of engineered systems.

Critically understanding the transition states and energy barriers of engineered doped nanoclusters is crucial for evaluating their catalytic behavior, particularly regarding encapsulation, doping, and metal doping^59^. Figures 8 and 9 present the transition state geometries and corresponding energy barriers for the designed surfaces. The chemisorption of atomic and molecular hydrogen was most significant on metal-encapsulated and doped surfaces, with minimal geometric changes observed, indicating that these modifications had little effect on hydrogen formation and evolution. Carbon atoms and transition metals were identified as key catalytic sites for hydrogen evolution, suggesting that optimizing these metal centers could enhance H–H adsorption energy and improve HER efficiency.

Various strategies have been explored to boost HER activity in catalytic materials, as summarized in Table 4. Common methods include doping and co-doping, which modify electronic properties and create additional active sites. Defect engineering, which introduces structural defects, also increases active sites and facilitates water dissociation during HER. For example, Su et al. achieved low overpotentials using a ruthenium-cobalt bimetallic alloy in nitrogen-doped graphene^47^. Additionally, a borophene monolayer oxide (h-B₂O) demonstrated promising HER performance through ad-atom doping and defect engineering. More so, hybridization has shown potential as well; a study combining Co₃O₄ nanoparticles with a conductive polymer resulted in a threefold increase in HER activity. Hitherto, our stepwise approach, utilizing encapsulation, doping significantly enhanced the catalytic properties of transition metal-engineered silicon-based materials. Given the alignment of our results with experimental findings, we believe this method could guide future experimental investigations of H₂@B_x_^dop^Ni^dop^Au^enc^Si_59_ and similar systems for optimized HER performance.

**Table 4:** Comparative hydrogen evolution reaction (HER) activity of surfaces.

Electrocatalyst	Strategy	$\Delta G_{ads}^{H}$(eV)
H_2_@B_x_^dop^Ni^dop^Au^enc^Si_59_ (this work)	Encapsulation and doping	(− 0.837 to − 0.848)
Pristine h-B_2_O^45^	Strain engineering	− 0.07
N-Ti_2_C_2_O_2_. Mxene^47^	Doping	0.087
B- Ti_2_C_2_O_2_.^[^Li, mat A^47^	Doping	− 0.097
Co_2_P^48^	Facet Engineering	0.0756
N-Ti_3_C_2_O_2_^9^	Doping	− 0.058
Borophene^28^	Doping	− 0.35
Irn@graphene (n = 1, 5)^7^	Doping	− 0.61
B_3_S^10^	Nanosheet	− 0.30
W_1_N_1_C_3_^13^	Anchoring, Doping	0.033
Ni3Fe@N–C NT/NFs^25^	Electrospinning	− 0.14
MoP2^11^	Doping	0.20
Ni-CoP_3_^29^	Control engineering	− 0.13
Fe-Mo_2_C@NCF^30^	Doping	0.39
Ru/Co–N-C^32^	Chemical etching/doping	− 0.445

## Machine learning-driven insights into adsorption energies for HER

**Figure 10:**
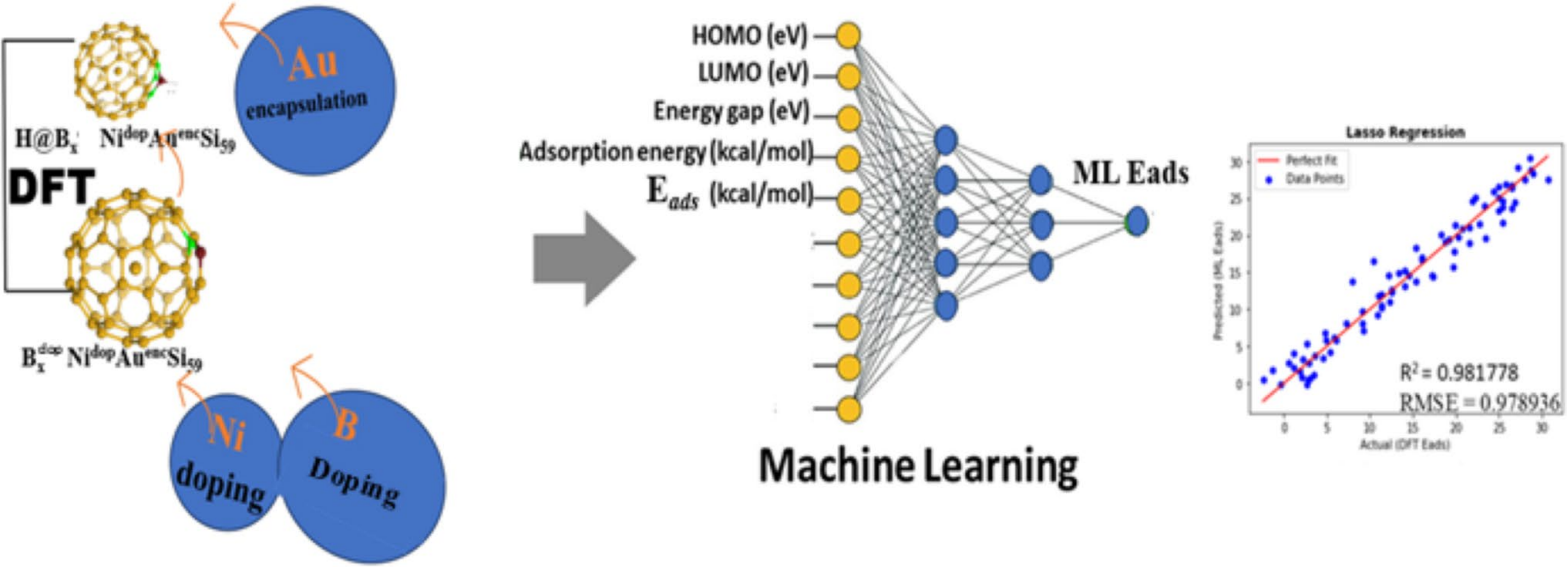
Pictorial illustration from DFT-engineered machine learning prediction.

**Figure 11:**
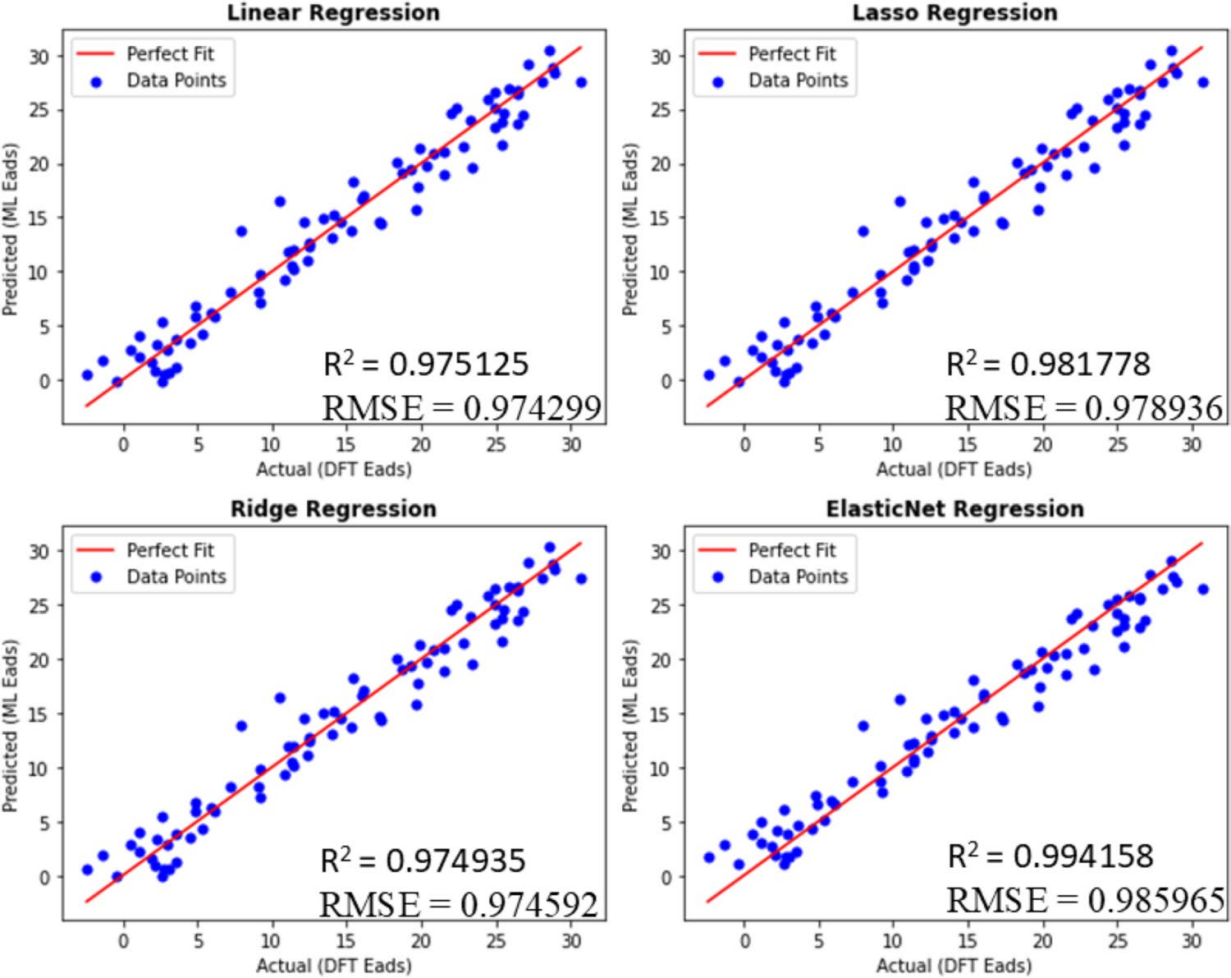
Comparison of DFT adsorption energies and ML predictions: high correlation and accuracy of regression models.

The machine learning (ML) models developed in this study (see Figs. 10 and 11) demonstrate a strong capability to predict adsorption energies (E_***ads***_) for hydrogen evolution reaction (HER) across various functionalized surfaces, ranging from H₂@Si₆₀ to H₂@B₃^dop^Ni^dop^Au^enc^Si_59_ systems. These predictions are based on regression models, specifically, Linear Regression, Lasso Regression, Ridge Regression, and ElasticNet Regression—each showing robust correlations between predicted and density functional theory (DFT)-calculated adsorption energies.

Firstly, with the H₂@Si₆₀ surface, the calculated adsorption energy of − 0.587 eV indicates a moderate interaction between hydrogen and the silicon-based surface. This relatively less negative value suggests weaker adsorption, which may hinder HER efficiency, as optimal surfaces require a balance between strong binding and the ease of hydrogen desorption. In contrast, the H₂@Au^enc^Si_60_ surface shows a slight improvement in adsorption energy to − 0.597 eV, indicating that gold functionalization enhances hydrogen adsorption, although the difference remains marginal. Thus, the H₂@Ni^dop^Au^enc^Si_59_ surface exhibits an even better performance with an adsorption energy of − 0.603 eV, highlighting nickel’s ability to form stronger interactions with hydrogen, making it a more promising candidate for HER. The ML models effectively capture this trend, as evidenced by high R^2^ values and low root mean square error (RMSE) scores across all regression methods, reinforcing their robustness in predicting subtle differences in adsorption energy. As such, further improvements are noted in the H₂@B_1_^dop^Ni^dop^Au^enc^Si_59_ and H₂@B_2_^dop^Ni^dop^Au^enc^Si_59_ systems, with adsorption energies of − 0.601 eV and − 0.598 eV, respectively. These boron-functionalized surfaces demonstrate stronger hydrogen binding compared to Si₆₀ and Au-based surfaces, though they are slightly less effective than the nickel-functionalized systems. The close alignment between predicted and actual adsorption energies for these systems underscores the ML models’ ability to resolve fine differences, validating their predictive capabilities.

Hence, the most promising surface identified is H₂@B_2_^dop^Ni^dop^Au^enc^Si_59_, which has probable adsorption energy, also indicating strong hydrogen adsorption and aligning well with optimal HER activity requirements. The combination of boron and additional functionalization results in superior performance, as the balance between hydrogen binding strength and reversibility is crucial for enhancing HER efficiency. Notably, the ElasticNet Regression model, with an R^2^ of 0.9942, accurately identifies the hydrogenated-boron systems as potent-performing surfaces. ElasticNet’s integration of Lasso and Ridge regularization using Eqs. 6–8 ensures the selection of the most relevant features while maintaining high predictive accuracy, making it a reliable tool for distinguishing efficient HER candidates.

6 $$y = \beta 0 + \beta 1x1 + \beta 2x2 + \cdots + \beta nxn + \varepsilon + \alpha \left| {\beta 1, \beta 2 \ldots \beta n} \right|$$

7 $$y = \beta 0 + \beta 1x1 + \beta 2x2 + \cdots + \beta nxn + \varepsilon + \alpha (\beta 1, \beta 2 \ldots \beta n) ^{2}$$

8 $$y = \beta 0 + \beta 1x1 + \beta 2x2 + \cdots + \beta nxn + \varepsilon + \alpha (\beta 1, \beta 2 \ldots \beta n) 2 + \alpha \left| {\beta 1, \beta 2 \ldots \beta n} \right|$$

In summary, the fine-tuned hydrogenated boron- doped systems emerge as a probable and potential systems due to their optimal adsorption characteristics, which effectively balance stability and reactivity. This trend highlights the significant impact of functionalization, particularly through boron-based modifications, which improve performance over Si₆₀, Au, and Ni surfaces. The results validate the role of machine learning in accelerating material screening for HER studies, providing highly accurate predictions with reduced computational effort compared to traditional DFT methods.

 Ultimately, the progression from H₂@Si₆₀ to H₂@B₃^dop^Ni^dop^Au^enc^Si_59_ and successfully identifying these promising systems as efficient surfaces underscore the power of machine learning in guiding experimental and computational research for energy applications. The strong predictive performance of all models ensures a reliable ranking of surfaces with optimal adsorption energies, positioning the hydrogenated engineered systems as potential and promising candidates for advancing HER technologies. While the machine learning (ML) models developed in this study were primarily used to predict hydrogen adsorption energies, their broader significance lies in capturing underlying structure–property relationships that govern catalytic behavior. The strong predictive performance of the ElasticNet model suggests that key descriptors, such as electronic structure parameters and compositional variations, are effectively encoded within the feature space. This framework can be extended toward rapid screening of catalytic candidates, optimization of doping configurations (e.g., boron concentration and distribution), and identification of trends in catalytic activity across compositional space. Such ML-assisted approaches complement density functional theory (DFT) by significantly reducing the computational cost associated with exhaustive configurational sampling, thereby accelerating catalyst discovery and optimization, Butler et al.^22^; Ramprasad et al.^60^. Beyond adsorption energy predictions, the present study can be interpreted within a descriptor-based framework for catalytic activity. In particular, the Gibbs free energy of hydrogen adsorption (ΔG_H_) serves as the primary activity descriptor for HER, with optimal catalytic performance achieved near thermoneutral conditions. In addition, electronic structure parameters obtained from DFT calculations, such as the HOMO–LUMO energy gap, electrophilicity index, and chemical hardness/softness, provide complementary descriptors that govern charge transfer, surface reactivity, and adsorption strength. The observed reduction in the energy gap and increase in electrophilicity upon boron and nickel co-doping indicate enhanced electronic flexibility and improved electron transfer capability, which are critical for efficient hydrogen adsorption and evolution. Notably, the B₂-doped system exhibits the most favorable balance of these descriptors, correlating with its near-optimal ΔG_H*_ value and suggesting an optimal doping configuration.

Within this context, the machine learning model developed in this work can be viewed as a tool for capturing and generalizing these descriptor–property relationships, enabling rapid screening of catalytic candidates and guiding the rational design of doped silicon-based nanoclusters for HER applications. Importantly, the integration of ML with DFT in this work provides a scalable pathway toward descriptor-based catalyst screening, aligning with emerging trends in data-driven materials discovery and accelerating the rational design of high-performance catalytic systems. Future work will focus on extending the ML framework toward descriptor discovery, optimization of dopant concentrations, and high-throughput screening of silicon-based nanostructures for enhanced HER performance.

## Conclusion

The geometric and electronic analyses confirm that hydrogen adsorption influences bond lengths in Ni-doped and B-doped Si₅₉ nanoclusters, indicating a stable and reactive catalytic environment. This study employed DFT to explore hydrogen adsorption on the Bx^dop^Ni^dop^Au^enc^Si₅₉ nanocluster, utilizing the B3LYP hybrid functional and SDD basis set for accurate electronic modeling, along with Grimme’s D3 correction for non-covalent interactions. The findings reveal that the introduction of boron significantly reduces the energy gap, with the B₂^dop^Ni^dop^Au^enc^Si₅₉ system achieving the smallest energy gap of 0.754 eV, indicating enhanced reactivity and improved HER performance. Enhanced hydrogen adsorption capabilities, particularly in the H₂@B₂^dop^Ni^dop^Au^enc^Si₅₉ system, show the vital role of boron doping in achieving superior catalytic performance. Furthermore, the results highlight that the adsorption energy calculations support the stability of the systems, and the Gibbs free energy assessments indicate that structured surface modifications effectively enhance catalytic activity for hydrogen production, emphasizing the importance of balancing energy gap fluctuations and electronic interactions to optimize the catalytic properties of engineered nanostructures. Among the Si_**59**_-based catalysts examined, H₂@B₂^dop^Ni^dop^Au^enc^Si₅₉ exhibited the most favorable hydrogen adsorption properties, with a Gibbs free energy change (ΔG_H_) of –0.836 eV, near the ideal thermodynamic value. This suggests a higher potential for hydrogen evolution reaction (HER) activity compared to the other configurations, making it a promising candidate for further development as an efficient HER electrocatalyst. The proposed B-doped Si₆₀ nanoclusters are not only theoretically promising but also experimentally realizable using current nanofabrication and doping techniques.

## Supplementary Information

Below is the link to the electronic supplementary material.

Supplementary Material 1.

## Data Availability

All data generated or analyzed during this study are included in this article and its supplementary information file**.**
