# Supplementary material for: Enhanced hydrogen adsorption on boron nickel gold modified Si60 nanocluster via DFT and machine learning analysis
Source: Sci Rep. 2026 May 19;16:22759. doi: 10.1038/s41598-026-52240-0 (PMC13385848; doi:10.1038/s41598-026-52240-0)
Supplement: Supplementary file 1 — Supplementary Material 1. [file 41598_2026_52240_MOESM1_ESM.docx]

**Enhanced hydrogen adsorption on boron nickel gold modified Si_60_ nanocluster via DFT and machine learning analysis.**

**Onyinye J. Ikenyirimba ^1*^, Gideon E. Mathias ^2,3*^, Chukwuma C. Nwanazoba ^4,5^, Anthony C. Iloanya^6^, Valentine Chikaodili Anadebe ^7^, Eno E. Ebenso^7^**

^1^ Department of Chemistry and Biochemistry, University of Arizona, Tucson, Arizona 85721, USA

^2^ Department of Pure and Applied Chemistry, Faculty of Physical Sciences, University of Calabar, Calabar, P.M.B 1115, Nigeria

^3^Biomedical Computational Chemistry Research Group, Lagos, Nigeria

^4^Department of Chemical Engineering, Auburn University, Alabama, 36849, USA

^5^Department of Chemical Engineering, Nnamdi Azikiwe University, P.M.B 5025, Awka, Nigeria.

^6^Department of Physics, Lehigh University, Pennsylvania, United States

^7^Centre for Materials Science, College of Science, Engineering and Technology, University of South Africa, Johannesburg 1710, South Africa

***Corresponding authors’ email:** [**mathiasgideon610@gmail.com**](mailto:mathiasgideon610@gmail.com)**,** [**ikenyirimbajoy@arizona.edu**](mailto:ikenyirimbajoy@arizona.edu)

**Table S1**. Calculated Second Order Perturbation Energies (ΔE Charge Transfer) and Diagonal Interactions Between Donor and Acceptor.

| Systems |  | Hybrid orbital | Transition | S-character (%) | P-character (%) |
| --- | --- | --- | --- | --- | --- |
| Si_60_ |  | SP | - | 29.043 | 70.956 |
| Au^enc^Si_60_ |  | SP | - | 28.977 | 71.022 |
| Ni^dop^Au^enc^Si_59_ |  | SP | - | 29.051 | 70.948 |
| B_1_^dec^Ni^dop^Au^enc^Si_59_ |  | SP | - | 29.047 | 70.952 |
| B_2_^dec^Ni^dop^Au^enc^Si_59_ |  | SP | - | 30.929 | 69.070 |
| B_3_^dec^Ni^dop^Au^enc^Si_59_ |  | SP | - | 28.966 | 71.033 |
| H_2_@ Si_60_ |  | SP^2^ | SP→SP^2^ | 29.034 | 70.965 |
| H_2_@Au^enc^Si_60_ |  | SP^2^ | SP→SP^2^ | 28.969 | 71.030 |
| H_2_@Ni^dop^Au^enc^Si_59_ |  | SP^2^ | SP→SP^2^ | 29.036 | 70.963 |
| H_2_@B_1_^dec^Ni^dop^Au^enc^Si_59_ |  | SP^3^ | SP→SP^3^ | 29.040 | 70.959 |
| H_2_@B_2_^dec^Ni^dop^Au^enc^Si_59_ |  | SP^3^ | SP→SP^3^ | 30.922 | 69.077 |
| H_2_@B_3_^dec^Ni^dop^Au^enc^Si_59_ |  | SP^3^ | SP→SP^3^ | 28.976 | 71.023 |

**TableS2.** Hybridization and Deformation in the Studied Silicon-Based Systems with TM-Au, Ni Doping, and Boron After Hydrogen Adsorption.

**Table S3.** Topological Parameters of Bond Critical Points in Engineered Metal-Functionalized Systems from QTAIM Analysis.

**N:B:** All parameters: ρ(r),
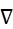
^2^ρ(r), V(r), K(r), G(r), ε, ELF, and H(r) are in a.u, respectively.

| **Systems interaction** | **Parameters** | **Bond (Kcal/mol)** | **p(r)** | **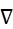^2^ρ(r)** | **G(r)** | **K(r)** | **V(r)** | **H(r)** | **G(r)/V(r)** | **ELF** | **Ɛ** | **λ_1_** | **λ_2_** | **λ_3_** | **λ_1/_ λ_3_** |
| --- | --- | --- | --- | --- | --- | --- | --- | --- | --- | --- | --- | --- | --- | --- | --- |
| H_2_@Si_60_ | BCPs |  | 0.213 | 0.619 | 0.152 | -0.188 | -0.150 | 0.188 | -1.013 | 0.846 | 0.299 | 0.916 | -0.129 | -0.167 | -5.485 |
|  | BE(EH_57—_Si_18)_ | -46 |  |  |  |  |  |  |  |  |  |  |  |  |  |
|  |  |  |  |  |  |  |  |  |  |  |  |  |  |  |  |
| H_2_@Au^enc^Si_60_ | BCPs |  |  |  |  |  |  |  |  |  |  |  |  |  |  |
|  | BE(ESi_22—_H_62)_ | -46 | 0.211 | 0.619 | 0.152 | -0.186 | -0.150 | 0.186 | -1.013 | 0.846 | 0.299 | 0.916 | -0.129 | -0.167 | -5.485 |
|  |  |  |  |  |  |  |  |  |  |  |  |  |  |  |  |
| H_2_@Ni^dop^Au^enc^Si_59_ | BCPs |  |  |  |  |  |  |  |  |  |  |  |  |  |  |
|  | BE(ENi_63—_H_61)_ | -47 | 0.217 | 0.636 | 0.183 | 0.240 | -0.207 | -0.240 | -0.884 | 0.654 | 0.468 | 0.898 | -0.106 | -0.155 | -5.793 |
|  | BCPs |  |  |  |  |  |  |  |  |  |  |  |  |  |  |
|  | BE(ENi_63—_Si_15)_ | -178 | 0.803 | 0.747 | 0.483 | 0.303 | -0.786 | -0.303 | -0.614 | 0.44 | 0.039 | -0.517 | 0.176 | -0.497 | 1.040 |
|  |  |  |  |  |  |  |  |  |  |  |  |  |  |  |  |
| H_2_@B_1_^dec^Ni^dop^Au^enc^Si_59_ | BCPs |  |  |  |  |  |  |  |  |  |  |  |  |  |  |
|  | BE(ENi_62—_H_60)_ | -47 | 0.217 | 0.627 | 0.181 | 0.240 | -0.205 | -0.240 | -0.882 | 0.671 | 0.508 | 0.899 | -0.108 | -0.163 | -5.515 |
|  | BCPs |  |  |  |  |  |  |  |  |  |  |  |  |  |  |
|  | BE(ENi_62—_Si_21)_ | -178 | 0.804 | 0.759 | 0.486 | 0.303 | -0.789 | -0.303 | -0.615 | 0.438 | 0.028 | -0.518 | 0.178 | -0.504 | 1.027 |
|  | BCPs |  |  |  |  |  |  |  |  |  |  |  |  |  |  |
|  | BE(ENi_62—_B_53)_ | -167 | 0.753 | -0.644 | 0.137 | 0.382 | -0.519 | -0.382 | -0.263 | 0.883 | 0.198 | -0.547 | 0.360 | -0.456 | 1.199 |
|  |  |  |  |  |  |  |  |  |  |  |  |  |  |  |  |
| H_2_@B_2_^dec^Ni^dop^Au^enc^Si_59_ | BCPs |  |  |  |  |  |  |  |  |  |  |  |  |  |  |
|  | BE(ENi_61—_H_58)_ | -51 | 0.232 | 0.667 | 0.193 | 0.263 | -0.219 | -0.263 | -0.881 | 0.727 | 0.469 | 0.966 | -0.177 | -0.121 | -7.983 |
|  | BCPs |  |  |  |  |  |  |  |  |  |  |  |  |  |  |
|  | BE(EB_62—_Ni_61)_ | -188 | 0.848 | 0.216 | 0.751 | 0.210 | -0.962 | -0.210 | -0.780 | 0.281 | 0.023 | -0.691 | 0.356 | -0.707 | 0.977 |
|  | BCPs |  |  |  |  |  |  |  |  |  |  |  |  |  |  |
|  | BE(EB_63—_Ni_61)_ | -189 | 0.855 | 0.213 | 0.746 | 0.214 | -0.960 | -0.214 | -0.777 | 0.289 | 0.022 | -0.697 | 0.354 | -0.712 | 0.978 |
|  |  |  |  |  |  |  |  |  |  |  |  |  |  |  |  |
| H_2_@B_3_^dec^Ni^dop^Au^enc^Si_59_ | BCPs | -179 | 0.807 | -0.106 | 0.123 | 0.408 | -0.531 | -0.408 | -0.231 | 0.924 | 0.149 | -0.611 | -0.531 | 0.768 | 0.795 |
|  | BE(ENi_60—_H_57)_ |  |  |  |  |  |  |  |  |  |  |  |  |  |  |
|  | BCPs |  |  |  |  |  |  |  |  |  |  |  |  |  |  |
|  | BE(ENi_60—_B_61)_ | -163 | 0.735 | 0.173 | 0.585 | 0.154 | -0.739 | -0.154 | -0.791 | 0.286 | 0.027 | -0.574 | 0.285 | -0.559 | 1.026 |
